# Chronic viral infection alters PD-1 locus subnuclear localization in cytotoxic CD8^+^ T cells

**DOI:** 10.1016/j.celrep.2024.114547

**Published:** 2024-07-30

**Authors:** Catarina Sacristán, Ben A. Youngblood, Peiyuan Lu, Alexander P.R. Bally, Jean Xiaojin Xu, Katelyn McGary, Susannah L. Hewitt, Jeremy M. Boss, Jane A. Skok, Rafi Ahmed, Michael L. Dustin

**Affiliations:** 1 Skirball Institute of Biomolecular Medicine, New York University School of Medicine, New York, NY, USA; 2 Emory Vaccine Center and the Department of Microbiology and Immunology, Emory University, Atlanta, GA, USA; 3 Immunology Department, St Jude Children’s Research Hospital, Memphis, TN, USA; 4 Department of Pathology, New York University School of Medicine, New York, NY, USA; 5 The Kennedy Institute of Rheumatology, Nuffield Department of Orthopedics, Rheumatology and Musculoskeletal Sciences, University of Oxford, Oxford, UK

**Keywords:** CD8^+^ T cell, cytotoxic, PD-1, exhaustion, chronic viral infection, subnuclear localization, transcription, methylation, Blimp-1, L-selectin

## Abstract

During chronic infection, virus-specific CD8^+^ cytotoxic T lymphocytes (CTLs) progressively lose their ability to mount effective antiviral responses. This “exhaustion” is coupled to persistent upregulation of inhibitory receptor programmed death-1 (PD-1) (*Pdcd1*)—key in suppressing antiviral CTL responses. Here, we investigate allelic *Pdcd1* subnuclear localization and transcription during acute and chronic lymphocytic choriomeningitis virus (LCMV) infection in mice. *Pdcd1* alleles dissociate from transcriptionally repressive chromatin domains (lamin B) in virus-specific exhausted CTLs but not in naive or effector CTLs. Relative to naive CTLs, nuclear positioning and *Pdcd1*-lamina dissociation in exhausted CTLs reflect loss of *Pdcd1* promoter methylation and greater PD-1 upregulation, although a direct correlation is not observed in effector cells, 8 days post-infection. Genetic deletion of B lymphocyte-induced maturation protein 1 (Blimp-1) enhances *Pdcd1*-lamina dissociation in effector CTLs, suggesting that Blimp-1 contributes to maintaining *Pdcd1* localization to repressive lamina. Our results identify mechanisms governing *Pdcd1* subnuclear localization and the broader role of chromatin dynamics in T cell exhaustion.

## Highlights

• *Pdcd1* alleles dissociate from repressive nuclear lamina in exhausted LCMV-specific CTLs
• Conversely, *Sell* associates with repressive lamina in exhausted LCMV-specific CTLs
• Loss of *Pdcd1*-lamina association reflects increased PD-1 expression and loss of methylation
• Chronic LCMV exhaustion is coupled to increased Blimp-1 protein binding to *Pdcd1*

## Graphical abstract

**Figure:**
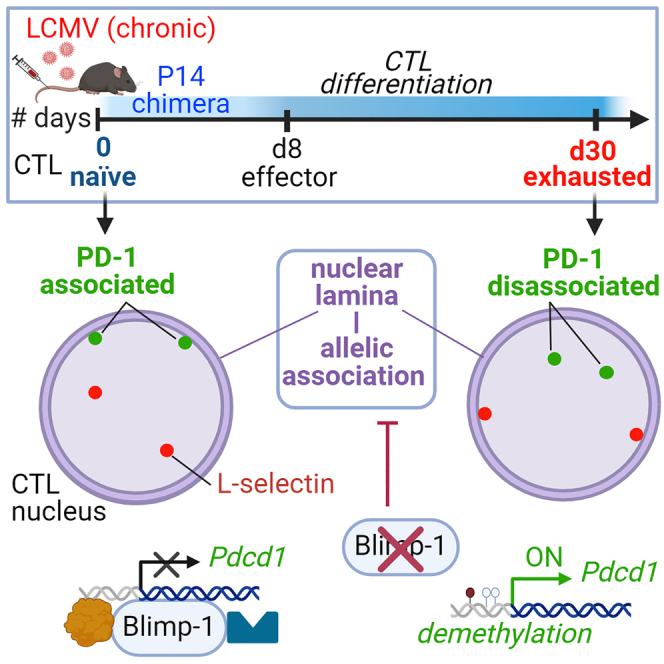

## Teaser

Sacristán et al. demonstrate that during mouse chronic viral infection, PD-1 alleles dissociate from transcriptionally repressive nuclear lamina in exhausted CD8^+^ T lymphocytes. Such allelic dissociation in exhausted cells accompanies upregulated PD-1 expression and promoter demethylation. Blimp-1 deficiency enhances *Pdcd1*-lamina dissociation, suggesting a mechanism for transcriptional modulation linked to chromatin dynamics.

## Introduction

Adaptive immunity balances the benefits of pathogen eradication against tissue injury resulting from potentially lethal lymphocyte clonal expansion and potent effector mechanisms.^1^ Implicated in immune tolerance,^2^^,^^3^ the programmed death-1 (PD-1) receptor contributes to maintaining healthy immune responses, keeping T cell activation “in check,” and protecting against immunopathology.^3^^,^^4^ With T cell receptor (TCR) activation, PD-1 expression is rapidly induced *in vivo*, and with sustained TCR stimulation (e.g., chronic infection), PD-1 expression is dramatically increased in CD8^+^ cytotoxic T lymphocytes (CTLs), dampening their killer activity.^5^^,^^6^^,^^7^ Such immune exhaustion^5^^,^^6^ is functionally defined by proliferative defects in memory stem cells and attenuated CTL effector functions,^8^^,^^9^^,^^10^^,^^11^ suppressing their attack to tolerate insults such as chronic infection.^9^^,^^12^^,^^13^^,^^14^ Indeed, exhaustion facilitates the persistence of various viruses and tumors.^12^^,^^15^^,^^16^^,^^17^^,^^18^ Although the suboptimal proliferation and effector function of exhausted CTLs is partly due to increased PD-1 gene and protein expression,^6^^,^^9^ the precise mechanisms mediating changes in PD-1 transcriptional regulation during CTL differentiation into exhaustion remain incompletely understood.

Nuclear organization and chromatin positioning are widely accepted mechanisms of transcriptional regulation. Mounting evidence across species, including mammals, demonstrates that the nuclear periphery can act as a transcriptionally repressive compartment.^4^^,^^19^^,^^20^^,^^21^^,^^22^^,^^23^^,^^24^^,^^25^^,^^26^^,^^27^^,^^28^^,^^29^ Gene silencing at the perinuclear lamina is well recognized but can also occur within pericentromeric heterochromatin, as reported for *Igh* and *Tcr* loci in mouse lymphocytes.^30^^,^^31^ At the nuclear periphery, ubiquitously expressed type B lamins bind chromatin as well as chromatin-modifying proteins and transcriptional regulators.^22^^,^^24^^,^^26^^,^^32^^,^^33^^,^^34^^,^^35^ Numerous studies report silencing of genes tethered to inactive chromosomal regions of the nuclear lamina or lamina-associated domains (LADs).^26^^,^^36^^,^^37^ By contrast, movement of genes^4^^,^^28^^,^^38^^,^^39^ and transgenes away from LADs into the nucleoplasm has been associated with transcriptional activation in various organisms.^25^^,^^32^^,^^40^ Consequently, nuclear organization represents an important means of allelic modulation and cellular programming.

We hypothesized that nuclear positioning and transcriptional programming/modulation of *Pdcd1* (encoding PD-1) might accompany virus-specific T cell differentiation toward exhaustion. According to this model, if genes such as *Pdcd1* are positioned away from subnuclear repressive compartments such as LADs in CTLs, allelic disassociation could be coupled to transcriptional activation in a high percentage of cells.^29^^,^^41^ Conversely, if a gene is silenced, a high percentage of CTLs might exhibit allelic association to the nuclear lamina. Relative to other CTL differentiation states, a higher number of cells with biallelic disassociation from the lamina could reflect modulated gene activation upon exhaustion, as surmised for highly upregulated *Pdcd1* during CTL exhaustion.^42^^,^^43^^,^^44^ Reflecting aspects of this model, here, we provide imaging and transcriptional evidence of *in vivo-*altered *Pdcd1* nuclear positioning and chromatin dynamics during CTL exhaustion in lymphocytic choriomeningitis virus (LCMV)-infected mice.

## Results

### *Pdcd1* dissociates from repressive nuclear lamina in exhausted LCMV-specific CD8^+^ T cells

To investigate additional mechanisms regulating the induction and maintenance of *Pdcd1* expression during CTL differentiation and exhaustion, we interrogated *Pdcd1* locus positioning relative to transcriptionally repressive subnuclear lamina in CTLs following acute (Armstrong strain [Arm]) or chronic (Clone 13 [Cl13]) LCMV infection. Initial findings of allelic association to LADs led us to limit our analysis to nuclear lamin B (excluding pericentromeric heterochromatin). Concomitantly, we assessed *Pdcd1* transcriptional regulation during corresponding CTL differentiation stages.

**Figure 1:**
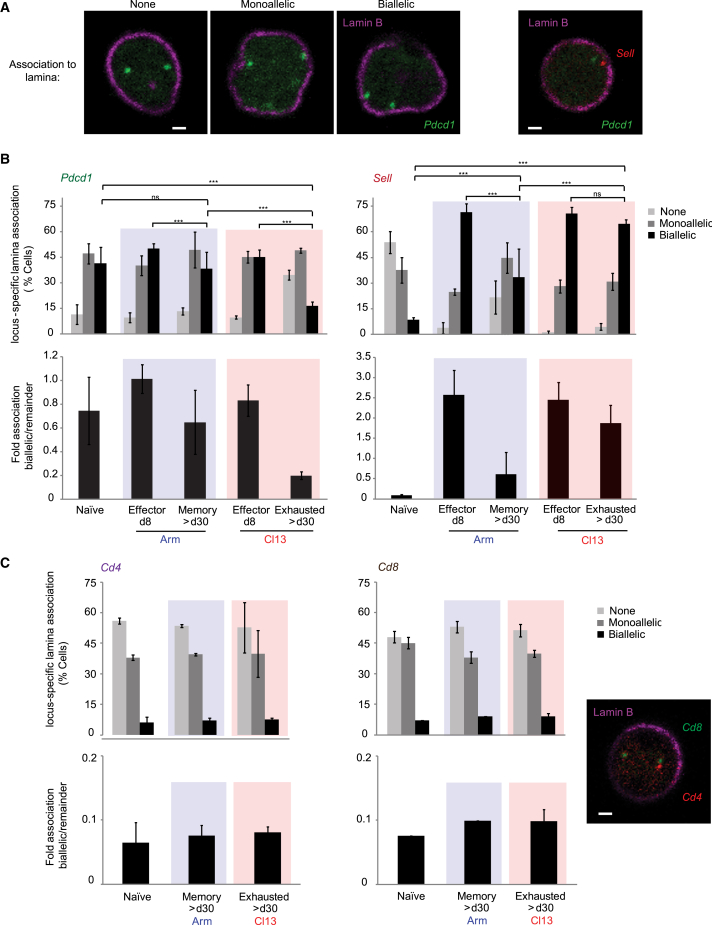
Exhausted antigen-specific CD8^+^ T cells lose biallelic *Pdcd1* association to repressive nuclear lamina (A) Representative confocal microscopy images show DNA-immunoFISH scoring examples of *Pdcd1* (green) and *Sell* (red) allelic association to lamin B (magenta) in LCMV-specific mouse effector CD8^+^ T lymphocytes (CTLs) as shown in (B). Left, middle, and right panels denote conditions of no association (none), monoallelic, or biallelic association to lamin B, respectively (specific focal planes shown). The far right panel displays an overlay example of *Sell* and *Pdcd1* loci in one single cell. Scale bars represent 1 μm. (B) LCMV-specific CTLs from Arm (blue) or Cl13 (red) were obtained at denoted days post-infection (dpi). Top graphs: the frequencies of cells with *Pdcd1* (left) or *Sell* (right) monoallelic or biallelic locus-specific association to lamin B (DNA-immunoFISH) are shown (% cells). Bottom graphs: *Pdcd1* (left) or *Sell* (right) biallelic fold association to lamina was calculated relative to the remaining conditions (none + monoallelic). (C) Negative controls for experiments presented in (A) and (B) are shown. Top graphs: the frequency of cells with *Cd4* (left) or *Cd8* (right) monoallelic or biallelic locus-specific association to lamin B (DNA-immunoFISH) (% cells) is shown. Bottom graphs: *Cd4* (left) or *Cd8* (right) biallelic fold association to lamin B was calculated as in (B). The far right panel displays a representative overlay confocal microscopy image of *Cd4* and *Cd8* loci in a single cell. (A–C) Independent experiments were reproduced ≥ *n* = 2–3 times; *n* = 3 mice per condition, per experiment. FISH ≥100 cells. *p* values: ns, not significant; ^∗^, significant; ^∗∗^, very significant; ^∗∗∗^, highly significant (see STAR Methods). Significance was calculated across all groups (Figures S1 and S2; Tables S1, S2, and S3). Graphs, *p* values combine 2–3 independent, representative experiments. (C) Differences in *p*, not significant. Errors bars = values ± SD.

Using three-dimensional DNA fluorescence *in situ* hybridization (3D-DNA-FISH; immunoFISH) and confocal imaging, we quantitatively scored *Pdcd1* in nuclei from sorted LCMV-specific CTLs derived from spleens of acutely or chronically infected C57BL/6 Thy1.2^+^ mice harboring Thy1.1^+^ P14 transgenic T cells (controlling TCR specificity and affinity differences) (Figures 1A, 1B, S1A, and S1B; Table S1). We dissected the percentage of P14 CTLs presenting “monoallelic,” “biallelic,” or “no allelic” association to lamin B (monoallelically expressing cells tend to have lower transcript amounts than biallelically expressing cells^45^). Generally, biallelic association to lamina suggests strong transcriptional repression, whereas no association reflects a transcriptionally permissive status^30^ (Figures 1A and 1B).

**Figure 2:**
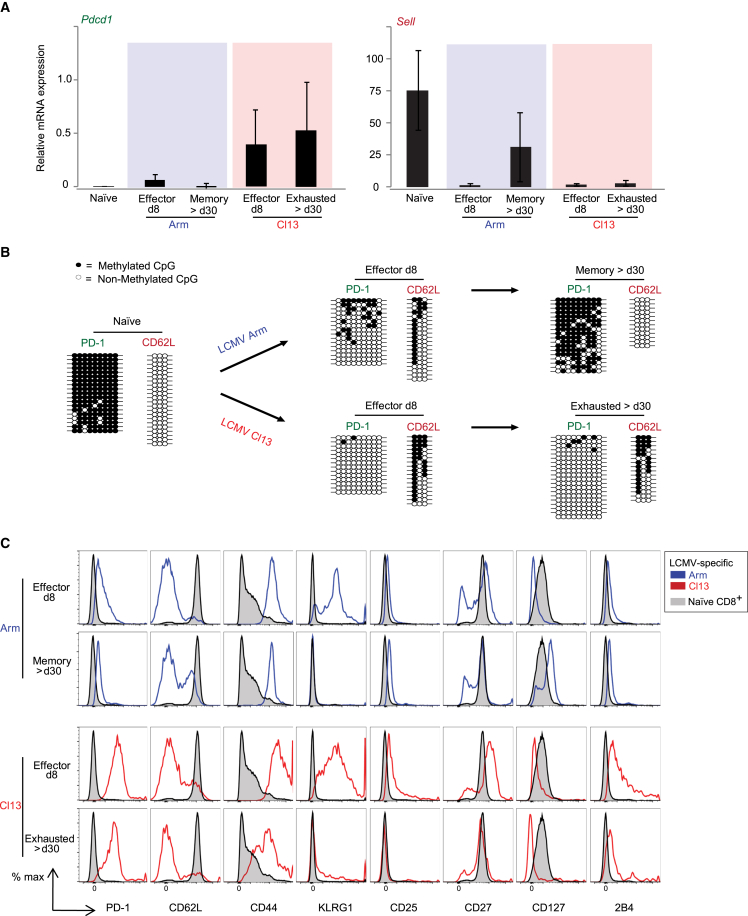
Loss of biallelic *Pdcd1*-lamina association in exhausted antigen-specific CD8^+^ T cells is consistent with increased PD-1 mRNA and protein, as well as DNA demethylation (A–C) LCMV-specific CD8^+^ T lymphocytes (CTLs) from Arm- (blue) or Cl13-infected mice (red) were obtained at denoted days post-infection (dpi) (constituting the same samples and experiments shown in Figure 1). Representative and independent experiments were reproduced ≥ *n* = 3; *n* = 3 mice per condition, per experiment. (A) The graphs denote relative mRNA expression of *Pdcd1* (left) or *Sell* (right) in CTLs. Error bars = values ± SD. (B) Results from genomic DNA bisulfite sequencing of *Pdcd1* and *Sell* promoter regions are shown. Lines: individual sequenced clones. Filled and open circles: methylated and nonmethylated cytosines, respectively. (C) Shown are FACS histograms of PD-1, CD62L, CD44, KLRG1, CD25, CD27, CD127, and 2B4 protein expression from samples in (A) and (B) (Figures 1, S1, and S2).

During chronic infection, exhausted P14 CTLs from ≥30 days post-infection (dpi) Cl13 mice (with elevated PD-1 mRNA/protein^42^^,^^43^) exhibited a significant loss (lower percentage of cells) of locus-specific *Pdcd1* biallelic association to repressive nuclear lamina relative to naive cells (and to 8-dpi effector cells) and relative to 30-dpi CTLs from Arm-infected mice (memory phenotype) (Figures 1A and 1B; Table S1). The fold association of cells exhibiting biallelic association to lamina relative to the remaining cells is also shown for easier visualization, highlighting the marked reduction in *Pdcd1*-lamina association in exhausted cells relative to naive or 8-dpi effector cells (approximately 2.5-fold and 3-fold decreases, respectively) (Figure 1B, lower panel). Lamina dissociation in exhausted cells accompanied high *Pdcd1* mRNA (Figure 2A, left) and PD-1 protein expression (Figure 2C), as well as loss of repressive CpG site-DNA methylation in the *Pdcd1* locus (Figure 2B^43^). However, elevated PD-1 mRNA/protein in Cl13 8-dpi effector CTLs did not correlate with *Pdcd1* nuclear positioning, which was unexpected.

During Arm infection, naive, effector 8-dpi, and memory ≥30-dpi LCMV-specific P14 CTLs harbored a high percentage of biallelic *Pdcd1*-lamin B associations (Figures 1A and 1B, left), suggesting low PD-1 gene/protein expression (Figures 2A, left, and 2C) and high DNA methylation (Figure 2B) in memory CTLs. Surprisingly, relative to naive cells, in Arm 8-dpi effector cells, higher PD-1 mRNA/protein expression (Figures 2A, left, and 2C) and lower DNA methylation (Figure 2B) did not correlate with *Pdcd1* nuclear positioning since these cells retained high allelic association to lamina, warranting further investigation.

Of note, high splenic and peripheral viral titers were confirmed at ≥30 dpi (consistent with earlier work ^43^^,^^46^ and data not shown). Fluorescence-activated cell sorting (FACS) phenotypic profiles of CTL differentiation subsets used in all assays corroborated surface protein changes during infection (e.g., PD-1, CD62L, CD44, KLRG1, CD25, CD27, CD127, and 2B4) (Figure 2C and data not shown), consistent with previously reported profiles of naive, effector, memory, and exhausted CTLs.^42^^,^^47^ Functional analysis (intracellular staining/FACS) confirmed impaired production of factors such as interferon γ (IFNγ), tumor necrosis factor alpha (TNF-α), interleukin-2 (and others) in exhausted CTLs relative to other cell subsets^6^ (Figure S2). Collectively, our findings suggest that during acute infection, *Pdcd1* alleles retain a relatively high association to lamina in memory CTLs compared to naive cells. Importantly, during chronic infection, loss of *Pdcd1* association to nuclear lamina correlates with permissive gene expression in exhausted CTLs relative to naive cells, albeit not in 8-dpi CTLs.

### L-selectin associates with repressive nuclear lamina in exhausted LCMV-specific CD8^+^ T cells

For comparison purposes and to control for locus nuclear localization in CTL populations, we simultaneously performed the same positioning and transcriptional analysis on L-selectin (*Sell*)–a gene encoding an adhesion molecule (surrogate marker of CTL memory differentiation and homing^47^^,^^46^) (Figures 1A and 1B, right, S1A, and S1B; Table S2). Compared to *Pdcd1*, *Sell* exhibited an inverse pattern of association to lamin B in exhausted cells: in naive cells, *Sell* loci were minimally associated to nuclear lamina and exhibited a significantly elevated expression of *Sell* mRNA/CD62L protein (Figures 2A, right, and 2C) as well as DNA demethylation of the *Sell* locus proximal CpG *de novo* methylation site^48^ (Figure 2B); by contrast, *Sell* loci were significantly associated to lamina in exhausted cells, coinciding with low *Sell* mRNA/CD62L expression (Figures 2A, right, and 2C), as well as with *Sell* DNA methylation (Figure 2B). The percentage of cells exhibiting biallelic *Sell*-lamina association increased 7.5- to 8-fold in exhausted cells relative to naive cells (Figure 1B). During acute infection, the percentage of 30-dpi memory CTLs presenting biallelic *Sell-*lamina association was 3.3-fold lower than in 8-dpi effector CTLs (Figure 1B, right), consistent with higher L-selectin mRNA/protein expression and increased *Sell* demethylation in memory CTLs (Figures 2A–2C). The data demonstrate that changes in *Sell* expression are significantly associated with changes in locus positioning in LCMV-specific CTLs, during both acute and chronic infections.

### *Cd4* and *Cd8* associations to nuclear lamina in LCMV-specific CD8^+^ T cells are invariant during acute or chronic infections and are mainly monoallelic

To further examine the link between T cell exhaustion and changes in nuclear positioning of *Pdcd1* and *Sell* loci, we assessed T-lineage-specific loci *Cd4* and *Cd8*.^49^^,^^50^ ImmunoFISH demonstrated that *Cd4* and *Cd8* associations to lamin B were generally monoallelic. Importantly, these associations were comparable in naive, memory (Arm), and exhausted (Cl13) CTLs. These findings indicated that unlike *Cd4* and *Cd8* control loci, changes in *Pdcd1* and *Sell* subnuclear localization were truly dependent on CTL differentiation status and type of LCMV infection (acute vs. chronic) (Figures 1C, S1A, and S1B; Video S1; Table S3).

Video S1. Biallelic positioning of Cd4 and Cd8 loci in an LCMV-specific CTL, related to Figure 1

### Chronic LCMV-mediated exhaustion is coupled to increased Blimp-1 transcription factor binding to *Pdcd1*

**Figure 3:**
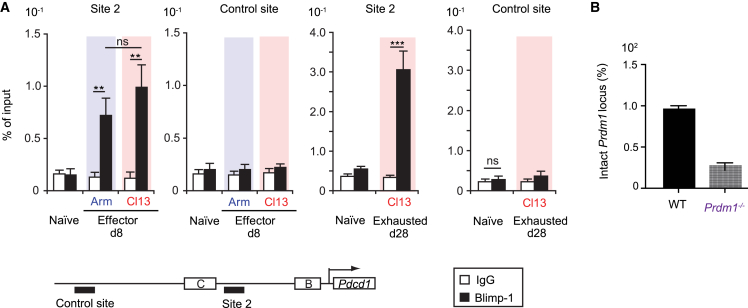
Chronic LCMV-mediated exhaustion is associated with increased Blimp-1 transcription factor binding to *Pdcd1* in CD8^+^ T cells LCMV-specific CD8^+^ T lymphocytes (CTLs) were obtained from Arm- (blue) or Cl13-infected mice (red) or wild-type (WT) C57BL/6 mice. CTLs from infected mice were collected at 8 dpi, as well as at 28 dpi for Cl13 mice. (A) ChIP analysis shows Blimp-1 binding to site 2 of the *Pdcd1* promoter and a control site in CTLs. Results are presented as percentage of input DNA. 8- and 28-dpi experiments were performed separately. 28-dpi samples combined cells from 5 mice each. Data were averaged from 3 independent experiments. *p* values: ns, not significant; ∗, significant; ∗∗, very significant; ∗∗∗, highly significant (see STAR Methods). (B) The graph denotes the quantitation of intact *Prdm1* locus by real-time PCR on DNA from Cl13 Blimp-1 conditional KO (*Prdm1*^*−/−*^) and WT control CTLs used in Figures 4, S1C, and S3. (A) and (B) Error bars = values ± SD.

To explore a possible mechanism for changes in *Pdcd1* nuclear positioning during T cell exhaustion, we tested whether known transcriptional regulators modulated allelic dissociation from nuclear lamina. The transcription factor B lymphocyte-induced maturation protein 1 (Blimp-1)—a master regulator of CD8^+^ T cell terminal differentiation^51^^,^^52^^,^^53^^,^^54^—is implicated in epigenetically associated repression of PD-1.^55^^,^^56^ 8-dpi and 30-dpi CTLs from Arm and Cl13 LCMV-infected mice both express Blimp-1, although Cl13 CTLs exhibit higher mRNA/protein expression than Arm CTLs.^55^ During acute infection, Blimp-1 conditional knockout (KO) mice (*Prdm1*^*−/−*^) exhibit sustained PD-1 expression in 8-dpi virus-specific CTLs relative to wild-type (WT) mice.^56^ Blimp-1 represses PD-1 expression in *in vitro* reporter assays at an early effector CTL stage, suggesting that it plays a role in regulating *Pdcd1* gene expression.^55^^,^^56^ Moreover, during acute infection of WT mice 8 dpi, chromatin immunoprecipitation (ChIP) assays show increased Blimp-1 binding to *Pdcd1* relative to naive cells at site 2 (between conserved region C [CR-C] and the *Pdcd1* transcription start site).^56^ We confirmed that Blimp-1 directly bound to the *Pdcd1* locus via ChIP in LCMV-specific 8-dpi WT CTLs (acute or chronic) (Figure 3A). 8-dpi effector CTLs from Arm- or Cl13-infected mice showed a significant increase in Blimp-1 binding to the *Pdcd1* locus relative to naive CTLs (approximately 3.5- to 4-fold) (Figure 3A). This observation further corroborates recently reported ChIP data using non-transgenic acute LCMV 8-dpi CTLs.^57^ In our study, we also demonstrated Blimp-1 binding to the *Pdcd1* locus in 28-dpi Cl13-specific CTLs (28-dpi Arm memory CTLs were not tested). These data extend the aforementioned Blimp-1-*Pdcd1* ChIP findings^57^ to truly exhausted CD8^+^ T cells and suggest that Blimp-1-*Pdcd1* binding is sustained during exhaustion. Collectively, the results validate a significant regulatory role of Blimp-1 on *Pdcd1* gene expression in acute and chronic viral infection settings.

### Blimp-1-deficient LCMV-specific CD8^+^ T cells lose *Pdcd1* association to repressive nuclear lamina during viral infection

**Figure 4:**
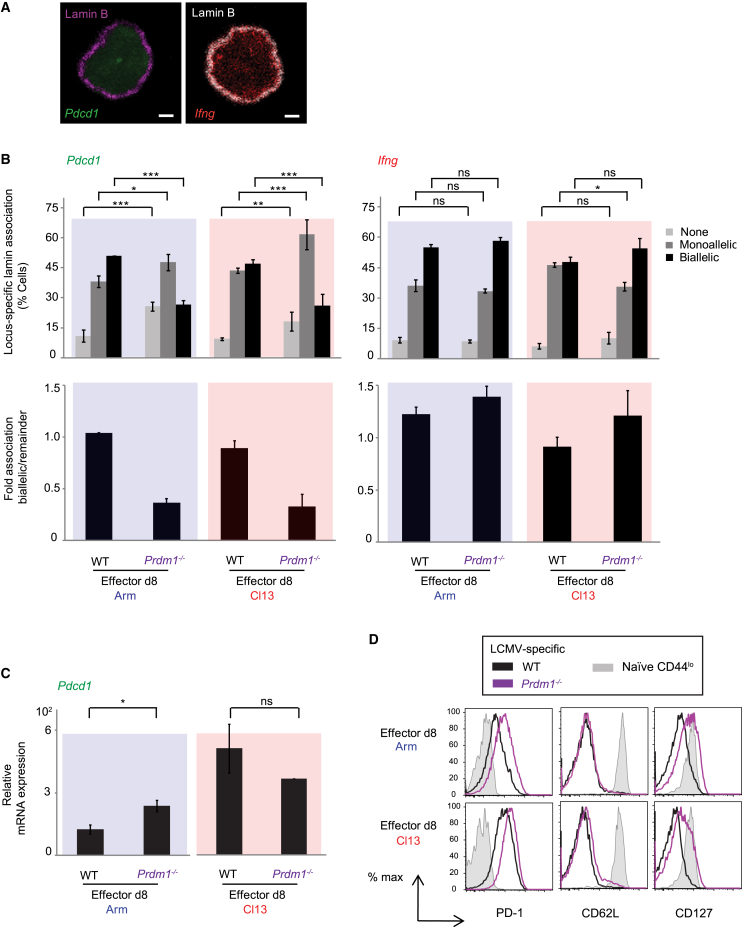
Blimp-1 modulates *Pdcd1* subnuclear localization to lamina in LCMV-specific effector CD8^+^ T cells LCMV-specific effector CD8^+^ T lymphocytes (CTLs) from Arm- (blue) or Cl13-infected (red) Blimp-1 conditional KO (*Prdm1*^*−/−*^) and wild-type (WT) mice were obtained at 8 days post-infection (dpi). Biallelic *Pdcd1* association to lamin B was scored via DNA-immunoFISH. Samples corresponding to Figures 4A and 4B are shown. (A) Representative confocal microscopy images depict DNA-immunoFISH examples of *Pdcd1* (green) and *Ifng* (negative control, red) allelic association to lamin B (magenta) in WT and *Prdm1*^*−/−*^ LCMV-specific mouse effector CTLs described in (B). Scale bars represent 1 μm. (B) Top graphs: the frequencies of cells with *Pdcd1* (left) or *Ifng* (right) no association (none), monoallelic, or biallelic locus-specific association to lamin B (DNA-immunoFISH) are shown (% cells). Bottom graphs: *Pdcd1* (left) or *Ifng* (right) biallelic fold association to lamina was calculated relative to the remaining conditions (remainder = none + monoallelic). (C) Relative mRNA expression of *Pdcd1* (left) or *Sell* (right) from samples in (A) and (B) is shown. (D) Shown are FACS histograms of PD-1, CD62L, and CD127 protein expression from samples in (A) and (B). Blue, *Prdm1*^*−/−*^; red, WT. Independent experiments were reproduced ≥ *n* = 2–3 times; *n* = 3 mice per condition, per experiment. FISH ≥100 cells. *p* values: ns, not significant; ^∗^, significant; ^∗∗^, very significant; ^∗∗∗^, highly significant (see STAR Methods). Significance was calculated across all groups (Tables S4, S5, S6, and S7). Graphs, *p* values combine 2–3 independent, representative experiments. Errors bars = values ± SD. (See also Figures S1C, S3, and S4).

Having confirmed bona fide Blimp-1 binding to the *Pdcd1* locus in CTLs, we next interrogated whether Blimp-1 played a role in modulating the localization of *Pdcd1* alleles relative to repressive nuclear lamina. We calculated *Pdcd1*-lamin B associations in Arm or Cl13 virus-specific splenic 8-dpi effector CTLs from WT versus *Prdm1*^*−/−*^ mice^56^ (Figure S1C shows the FACS gating strategy). The degree of Blimp-1 *Cre* recombination in *Prdm1*^*−/−*^ mice (assessed via PCR) confirmed substantial ablation of the intact *Prdm1* locus in effector LCMV-specific CTLs (Figure 3B). Because differences in Blimp-1 binding to *Pdcd1* by ChIP (Figure 3A) were detected as early as 8 dpi in effector Arm or Cl13 CTLs, we performed our analyses using this time point. Importantly, this time point allowed us to obtain the minimal number of cells needed to successfully perform DNA-FISH experiments in *Prdm1*^*−/−*^ versus WT mice. Obtaining sufficient CTLs from *Prdm1*^*−/−*^ mice for DNA-FISH at the 28-dpi time point for Arm or Cl13 infections was unfortunately not feasible in these mutant mice. Nevertheless, the data showed that at 8 dpi, Blimp-1-deficient effector CTLs from chronically or acutely infected mice exhibited a significant loss in *Pdcd1* association to lamina relative to WT mice (Figures 4A and 4B, left; Tables S4 and S5). This was evidenced by a decreased percentage of biallelic *Pdcd1*-lamina association in *Prdm1*^*−/−*^ CTLs relative to WT CTLs (≥2-fold decrease in both acute and chronic conditions; Figure 4B, left). The fold association of cells exhibiting biallelic association (to lamina) relative to the remaining cells is also shown (Figure 4B, lower panel). These findings are consistent with previous data indicating that in the absence of Blimp-1, *Pdcd1* expression is not fully downregulated in early effector CTLs, and that during Arm infection, Blimp-1-deficient virus-specific CTLs exhibit sustained PD-1 expression from 8 to 28 dpi, compared to WT CTLs.^56^ We confirmed that in the Arm and Cl13 8-dpi CTL pools that were used for DNA-FISH, the accompanying PD-1 mRNA/protein expression was higher in *Prdm1*^*−/−*^ cells than in WT (Figures 4C and 4D). This was also consistent with acute LCMV infection relieving Blimp-1 repression of PD-1 in murine CTLs (at this time point).^56^ Of note, we also recapitulated previous findings of upregulated CD62L and CD127 in CTLs with Blimp-1 deletion during chronic infection^55^ in *Prdm1*^*−/−*^ CTLs relative to WT (Figure 4D). CD127 was also upregulated in Blimp-1-deficient Arm 8-dpi CTLs. Taken together, our results indicate that elevated PD-1 mRNA/protein in Arm and Cl13 8-dpi effector CTLs is consistent with decreased *Pdcd1* association to repressive lamina in the absence of Blimp-1.

As a DNA-FISH internal control in Blimp-1 deficiency experiments, we quantitated the association of *Ifng* alleles to nuclear lamina; we chose this control because the locus is known to constitutively localize to the nuclear periphery in CTLs^58^ (Figure 4B, right; Tables S6 and S7). This might initially seem counterintuitive because IFNγ secretion is lower in exhausted Cl13 CTLs compared with Arm CTLs^10^^,^^42^^,^^54^ (Figure S2). However, despite changes in IFNγ secretion between acutely and chronically infected mice, at 8 dpi (as well as during exhaustion), *Ifng* transcript expression is expected to occur.^42^^,^^59^ No difference in *Ifng* expression was observed between WT and *Prdm1*^−/−^ mice or between the two types of infection.^54^^,^^60^ As anticipated, the data showed that *Ifng*-lamina association was similar between WT and *Prdm1*^*−/−*^ cells for both types of infection. This validated bona fide impaired regulation of *Pdcd1* subnuclear localization in the absence of Blimp-1 in Cl13- and Arm-specific effector CTLs.

## Discussion

Our study highlights the importance of locus movement and nuclear positioning relative to transcriptional events and epigenetic programming in CD8^+^ T cells during viral infections *in vivo*. The results suggest that loss of *Pdcd1* association to repressive nuclear lamina is associated with the chronic phase of viral infection in exhausted CTLs (at 30 dpi). This loss is consistent with changes in gene accessibility (mRNA and methylation status) in exhausted CTLs.

However, the finding that biallelic *Pdcd1-*lamina association was maintained in 8-dpi Cl13 effector CTLs was surprising (Figure 1B). This occurred even when PD-1 mRNA/protein expression was elevated (Figure 2). Nevertheless, it should be noted that mRNA was quantitated from bulk CTLs per time point, not constituting biallelic vs. monoallelic mRNA representation/segregation in these cellular populations. Also, gene activation can occur within LADs,^61^ so this finding is not unprecedented. In addition, the locus positioning regulatory mechanism may be specific to later stages of chronic LCMV infection (and/or be context specific for infection type). Because 8-dpi CTLs—either from chronic or acute infections—harbored *Pdcd1* alleles that retained high association to lamina, it is likely that a specific transcriptional program in effector cells also depends on regulatory mechanisms other than those involving chromatin dissociation from lamina. Repositioning may not be a prerequisite for *Pdcd1* expression. Although the precise meaning of these findings in effector CTLs vs. other populations is difficult to determine, the unique modulation in *Pdcd1* nuclear positioning suggests ongoing changes and a distinct PD-1 configuration in LCMV-specific CTLs during infection.

Another plausible scenario is that there is a temporal component of *Pdcd1*-lamina disassociation and movement that is tightly linked to differentiation and programming of a CTL’s exhausted state. Because different time points represent only a snapshot of the cells in those moments, we posit that they may not fully reflect the dynamic physiological continuum that occurs in allelic nuclear positioning between 8 and 30 dpi. Indeed, evidence from similar LCMV infection models suggests that the exhaustion program begins rather early,^62^ with imprinted *Pdcd1* demethylation at the effector phase of CTL exhaustion, and it is sustained irreversibly thereafter.^63^ Therefore, assessing the 8-dpi effector time point remains an important approach. We propose that events such as allelic positioning and transcriptional regulation prepare CTL entry into a trajectory toward exhaustion. Consistent with studies documenting epigenetic changes during CTL exhaustion,^64^ we hypothesize that sometime between 8 and 30 dpi, there is a specific shift in biallelic positioning that dramatically tilts the status of *Pdcd1* transcription toward increased permissiveness and a fully exhausted state.

Also, our data suggest that the mechanisms regulating subnuclear localization of *Pdcd1* differ between acute and chronic LCMV infections (although this was not the main focus of our study). It is increasingly clear that the epigenetic profiles of exhausted CTLs widely differ from those of effector and memory cells, both in humans and mice.^65^^,^^66^^,^^67^^,^^68^

From another angle, *Pdcd1* transcriptional activation and loss of DNA methylation in 8-dpi CTLs may occur before allelic movement away from the lamina.^61^ Work from others has demonstrated that demethylation is not required for chromatin remodeling or primary transcription of a gene but might instead be a consequence of transcription, which is important for maintaining an active state.^69^ At face value, our data suggest that transcriptional activation could occur first, followed by demethylation, followed by stable movement away from the lamina. Of note, the unexpected observation of substantial methylation loss in Arm 8-dpi CTLs is interesting and merits further attention.

We do not claim that subnuclear *Pdcd1* localization is a dominant mechanism for repression or activation, but we posit that there is an evident and tangible change occurring at the exhausted stage that is linked to epigenetic reprogramming, which may not be reversible.^43^ Future work on the kinetics of *Pdcd1* chromatin dynamics may shed further light on this question.

Regarding *Sell* subnuclear positioning, we noted striking differences in locus association to repressive nuclear lamina across CTL states during both acute and chronic infections and compared to *Pdcd1*. During chronic infection, *Sell*-lamina associations were significantly higher in exhausted cells and effector cells compared to naive cells. Conversely, significant loss of *Pdcd1*-lamina associations was prominent in exhausted CTLs relative to naive cells. Overall, the data support a model of differential *Pdcd1* or *Sell* subnuclear allelic positioning impacting the gene regulatory network during CTL memory differentiation and exhaustion. Differential nuclear positioning of these loci is intriguing because it may be mediated by distinct mechanisms—a hypothesis that warrants further investigation.

When considering potential regulatory mechanisms, our results uncover a bona fide effect of Blimp-1 in modulating *Pdcd1* nuclear positioning in 8-dpi effector CTLs during acute and chronic infections. Blimp-1 deficiency leads to a loss of *Pdcd1*-lamina association, consistent with high PD-1 mRNA/protein expression. Blimp-1-mediated regulation of PD-1 expression may occur in part, but not exclusively, by influencing *Pdcd1* nuclear positioning. Upon chronic viral infection, tight control of PD-1 expression is expected in early effector cells; in the absence of Blimp-1, PD-1 repression may no longer occur, but the ensuing permissive effects on PD-1 expression may not be fully observed until a later time point, i.e., exhaustion.

An intriguing finding was that Blimp-1-deficient 8-dpi effector CTLs from chronically or acutely infected mice also exhibited an impaired ability to modulate *Sell* association to lamina relative to WT mice (Figure S3; Tables S8 and S9). This implicates Blimp-1 in modulating allelic subnuclear localization of both *Pdcd1* and *Sell* during chronic and acute LCMV infection. However, the significance of these findings and associated transcriptional outcomes remain to be thoroughly addressed.

We posit that transcription factors that regulate memory or exhaustion T cell fates might do so by modulating the nuclear positioning of genes (presumably not only by simply recruiting or blocking RNA polymerase II). Given the *Pdcd1* nuclear localization changes in *Prdm1*^*−/−*^ CTLs, one might predict changes in WT memory precursor CTLs vs. terminal effector CTLs. Accordingly, Blimp-1 deficiency in mice modifies the terminal effector vs. memory precursor ratios,^50^^,^^53^ which is a type of cellular kinetic response during differentiation; this type of transcriptional and epigenetic reprogramming is certainly complex at distinct time points during infection.

Also, because Blimp-1 deficiency did not completely ablate *Pdcd1*-lamina associations in effector CTLs, other regulators and transcription factors implicated in regulating T cell differentiation and exhaustion (e.g., T-bet, Eomes, Foxo1, Tcf-1, NFATc, TOX, LSD1, MYB, etc.)^52^^,^^57^^,^^62^^,^^64^^,^^70^^,^^71^^,^^72^^,^^73^^,^^74^^,^^75^ are likely implicated in changes in gene nuclear positioning and transcription and may act to coordinate chromatin accessibility of the *Pdcd1* locus. Accordingly, the enhancer-decommissioning, histone epigenetic eraser LSD1 contributes to Blimp1-mediated repression of *Pdcd1* expression in CTLs during acute LCMV infection^57^: Blimp-1 recruits LSD1, resulting in loss of all active histone modifications at the *Pdcd1* promoter regulatory regions. Although Blimp-1 binds during chronic infection, LSD1 is not recruited. Therefore, the selective recruitment of LSD1 during chronic vs. acute infection remains to be determined.

Factors such as these—cooperatively or separately—and/or other yet to be identified transcription factors and epigenetic regulators implicated in mammalian regulation of gene expression (e.g., H3K9 methyltransferases, DNA methyltransferases, and others) likely enable *Pdcd1* tethering to nuclear lamina proteins (e.g., anchors, DNA binding proteins, epigenetic readers, etc.).^26^^,^^35^ Indeed, we have evidence that loss of DNA methyltransferase Dnmt3a interferes with nuclear *Pdcd1*-lamina and *Sell*-lamina associations at 8-dpi effector CTLs during acute LCMV infection (Figure S4; Tables S10 and S11) (loss of biallelic associations); this was achieved by comparing WT vs. Dnmt3a conditional KO mice (*Dnmt3a*^−/−^).^48^^,^^64^ These findings suggest that Dnmt3a or its resulting activity (methylated DNA) might also contribute to influencing allelic nuclear positioning and programming. Accordingly, a previous report demonstrated that exhausted CTLs acquire a Dnmt3a-dependent *de novo* methylation program; in the absence of Dnmt3a, CTLs fail to acquire an exhausted phenotype.^64^

The role of various factors in allelic positioning relative to gene expression remains a broad, open question. Another consideration is that allelic positioning might affect the positioning/transcription of neighboring genes. Other than various regulators contributing to chromatin accessibility during allelic positioning, one possibility—not necessarily exclusive—is that our immunoFISH observations likely reflect a scenario in which not all alleles are transcriptionally active at any one given time—consistent with the dynamic kinetics of transcriptional activation, demethylation, and locus movement. Future causal experiments can provide mechanistic and functional insight that clarifies the kinetic interactions between these loci and specific repressive subnuclear domains. Overall, by taking an orthogonal approach to examine modulators of T cell differentiation during LCMV infection, our findings contribute to the growing body of evidence demonstrating that factors such as Blimp-1 (and Dnmt3a) are implicated in T cell differentiation and function and comprise a part of the broader gene regulatory network.

The relevance of our findings aligns with the broad interest to fully understand the molecular underpinnings of reversing T cell dysfunction, especially in the context of PD-1 regulation. The fields of tumor immunology, chronic infection (e.g., HIV-1 and hepatitis C virus), vaccinology, and autoimmunity are investigating the promising prospect of checkpoint blockade immunotherapy, largely based on encouraging results in treating certain cancers with anti-PD-1/PD-L1 antibodies. Here, CD8^+^ T cell exhaustion is reversed, converting this cell type into an effector killing machine.^76^^,^^77^^,^^78^^,^^79^^,^^80^ The implications of better understanding *Pdcd1* epigenetic regulation and chromatin dynamics in T cell differentiation and exhaustion are far-reaching because stable heritable epigenetic programming of exhausted CTLs in viral infections and tumors may limit targeting the PD-1/PDL-1 signaling pathway to reinvigorate these cells.^64^^,^^66^^,^^81^ However, blocking *de novo* Dnmt3a methylation enhances PD-1 blockade of tumor-infiltrating rejuvenated CTLs.^64^ Consequently, reinvigoration of CTLs will undoubtedly require precise fine-tuning, largely potentiated by the modulation of epigenetic states and chromatin accessibility. This underscores the importance of understanding nuclear domains and transcriptional regulation during memory T cell differentiation and exhaustion and may open interesting lines of investigation, including identifying putative therapeutic targets in infection, cancer, and autoimmunity.

### Limitations of the study

There may be nuances or variations in *Pdcd1*-specific chromatin dynamics, locus accessibility, and *Pdcd1* expression during effector-to-memory and effector-to-exhaustion differentiation, which are not readily explained by this brief study. Changes in these cell state transitions might be better visualized in specific CTL subpopulations,^42^^,^^47^^,^^82^ particularly in light of evidence describing the functional importance of memory-like Tcf-1^+^ CD8^+^ T cells harboring both central memory and exhausted T cell characteristics during chronic LCMV infection.^62^^,^^83^^,^^84^

We recognize that the lack of DNA-FISH and ChIP analyses of 28- to 30-dpi memory and exhausted CTL populations in *Prdm1*^*−/−*^ mice (insufficient available cells) prevents us from drawing definitive conclusions regarding the role of Blimp-1 in regulating PD-1 and L-selectin subnuclear positioning and transcriptional/epigenetic changes during chronic and acute LCMV infections. Causality for the dynamic interactions between Blimp-1 and PD-1 or L-selectin in repositioning and transcription are unlikely to be disentangled solely at the level of DNA-FISH and will require more sophisticated experimental designs.

## STAR★Methods

### Key resources table

**Table:** 

REAGENT or RESOURCE	SOURCE	IDENTIFIER
**Antibodies**		

BD Pharmingen™ PE Rat IgG2a, κ Isotype Control (Clone R35-95)	BD Biosciences	Cat # 553930
BD Pharmingen™ PE-Cy™7 Rat IgG2b, κ Isotype Control (Clone A95-1)	BD Biosciences	Cat # 552849
CD44-FITC anti-mouse (clone IM7)	BioLegend	Cat # 103006
CD44-APC anti-mouse/human (clone IM7)	BioLegend	Cat # 103012
CD44-Brilliant Violet 421(TM) anti-mouse/human (clone IM7)	BioLegend	Cat # 103039
CD8-FITC anti-mouse CD8a (clone 53-6.7)	BioLegend	Cat # 100705
CD8-PE anti-mouse CD8a (Clone 53-6.7)	BioLegend	Cat # 100707
CD8-APC anti-mouse CD8a (Clone 53-6.7)	BioLegend	Cat # 100711
CD8-PE-Cy7 anti-mouse CD8a (Clone 53-6.7)	BioLegend	Cat # 100721
CD8-PerCPCy5.5 anti-mouse CD8a (Clone 53-6.7)	BioLegend	Cat # 100733
CD8-PerCP anti-mouse CD8a (clone 53-6.7)	BioLegend	Cat # 100732
CD8-Pacific Blue anti-mouse CD8a (Clone 53-6.7)	BioLegend	Cat # 100728
Thy1.1-PE anti-rat CD90/mouse CD90.1 (Clone OX-7)	BioLegend	Cat # 202523
Thy1.1 Brilliant Violet 421™ anti-rat CD90/mouse CD90.1 (Clone OX-7)	BioLegend	Cat # 202535
Thy1.1-PerCP anti-rat CD90/mouse CD90.1 (Clone OX-7)	BioLegend	Cat # 202512
PD-1-PE/Cyanine7 anti-mouse CD279 (clone 29F.1A12)	BioLegend	Cat # 135216
PD-1-PE BD Pharmingen™ Hamster Anti-Mouse CD279 (clone J-43)	BD Biosciences	Cat # 561788
CD62L-FITC anti-mouse (clone MEL-14)	BioLegend	Cat # 104405
CD62L-PE anti-mouse (Clone MEL-14)	BioLegend	Cat # 104407
CD62L-APC anti-mouse (Clone MEL-14)	BioLegend	Cat # 104412
IFNγ-FITC anti-mouse (Clone XMG1.2)	BioLegend	Cat # 505805
KLRG1-FITC anti-mouse/human (Clone 2F1/KLRG1)	BioLegend	Cat # 138410
CD27-PE anti-mouse/human TNFR (CloneLG.3A10)	BioLegend	Cat # 124209
CD127-PE anti-mouse IL-7Rα (Clone S18006K)	BioLegend	Cat # 158203
CD25-PE anti-mouse IL-2Rα (CloneA18246A)	BioLegend	Cat # 113703
2B4-PE anti-mouse CD244.2 (2B4 B6 Alloantigen) (Clone m2B4 (B6)458.1)	BioLegend	Cat # 133507
CD69-PE anti-mouse (Clone H1.2F3)	BioLegend	Cat # 104507
Granzyme B-PE anti-mouse/human (Clone QA16A02)	BioLegend	Cat # 372207
TNFα-PE anti-mouse (Clone MP6-XT22)	BioLegend	Cat # 506305
IL-2-PE anti-mouse (Clone JES6-5H4)	BioLegend	Cat # 503807
IL-2-APC anti-mouse (Clone JES6-5H4)	BioLegend	Cat # 503809
Blimp-1 anti-mouse (polyclonal)	Rockland	Cat # 600-401-B52
Lamin B (C-20) goat polyclonal IgG, anti-mouse/human	Santa Cruz	Cat # sc-6216
Lamin B (M-20) goat polyclonal IgG anti-mouse/human	Santa Cruz	Cat # sc-6217
rabbit anti-mouse IgG	Millipore-Sigma	Cat # 06-371

**Bacterial and virus strains**		

Lymphocytic choriomeningitis virus, Armstrong strain, clone 53b	Rafi Ahmed laboratory, Matloubian et al.^85^	N/A
Lymphocytic choriomeningitis virus, Clone 13 strain	Rafi Ahmed laboratory, Matloubian et al.^85^	N/A
XL10-Gold ultracompetent *Escherichia coli* bacteria	Stratagene	Cat #200314
DH10 *Escherichia coli* clones	BACPAC Resources Online	https://bacpacresources.org

**Chemicals, peptides, and recombinant proteins**		

MHC class-I H2-D^b^ tetramer LCMV epitope GP33-APC	Yerkes NIH tetramer core facility, Emory University	N/A
MHC class-I H2-D^b^ tetramer LCMV epitope GP276-APC	Yerkes NIH tetramer core facility, Emory University	N/A
MHC class-I H2-D^b^ tetramer NP396 LCMV epitope	Yerkes NIH tetramer core facility, Emory University	N/A
18S ribosomal RNA	Applied Biosystems, ThermoFisher Scientific	Cat # 4308329
pGEM-T TA cloning vector	Promega	Cat#A3600
poly-L-lysine	Sigma	Cat# P4707
Electron microscopy grade 4% paraformaldehyde/0.1M PBS (pH 7–7.4)	Electron Microscopy Sciences	Cat# 15735-85
Triton X-100	Sigma	Cat #X100
RNaseA	Roche	Cat # 10109169001
Tween 20	Ambion	Cat# W3831
ProLong Gold/DAPI buffer	Invitrogen	Cat #P36931
Pierce™ ChIP-Grade Protein A/G Plus Agarose	ThermoFisher Scientific	Cat# 26161
ChromaTide Alexa Fluor 546-14-dUTP	Invitrogen	Cat# C11401
ChromaTide Alexa Fluor 568-5 dUTP	Invitrogen	Cat# C11399
ChromaTide Alexa Fluor 594-5 dUTP	Invitrogen	Cat# C11400
Cy3-dUTP	GE Healthcare, Amersham	Cat# PA55022
dTTP	GE Healthcare	Cat# 28406531
dNTP kit (dATP, dCTP, dGTP)	Sigma	Cat # 72004
DNase I recombinant	Roche	Cat # 04536282001
DNA polymerase I (E. coli)	New England Biolabs	Cat# M0209L
BSA Fraction V, OmniPur	EMD Chemicals	Cat# 2910
Formamide	Fisher Scientific	Cat# BP-227-500
Dextran sulfate	Sigma	Cat# D8906
50X Denhardt’s solution	Invitrogen	Cat# 750018
20x SSC	Ambion	Cat# AM9770
2-beta-mercaptoethanol	Sigma	Cat # M-3148
Pierce™ Protein A Magnetic Beads	ThermoFisher Scientific	Cat # 88845
Pierce™ ChIP-Grade Protein A/G Plus Agarose	ThermoFisher Scientific	Cat # 26161

**Critical commercial assays**		

GolgiPlug™ Protein Transport Inhibitor (containing Brefeldin A)	BD Biosciences	Cat # BDB555029
Cytofix/Cytoperm kit, BD	BD Biosciences	Cat# 554714
QIAGEN Plasmid Maxi Kit	QIAGEN	Cat # 12163
QIAGEN Plasmid Mini Kit (DNA purification)	QIAGEN	Cat # 12123
QIAGEN DNeasy kit	QIAGEN	Cat# 69506
QIAGEN RNeasy Kit	QIAGEN	Cat# 74104
EZ DNA methylation kit	Zymo Research	Cat# D5002
Taq polymerase–based PCR kit	QIAGEN	Cat# 201225
MACS CD8a+ T cell Isolation Kit II	Miltenyi Biotec	Cat# 130-095-236
MACS LS columns	Miltenyi Biotec	Cat# 130-042-401
MACS Pre-separation filters	Miltenyi Biotec	Cat# 130-095-823
PureLink HiPure Plasmid DNA Purification kit	Invitrogen	Cat# K2100-07

**Experimental models: Organisms/strains**		

Mouse: Transgenic D^b^GP33-41 TCR-tg P14 (P14)	Rafi Ahmed laboratory	N/A
Mouse: C57BL/6	Jackson Laboratory	JAX: 000664
Mouse: *Prdm1*^−/−^ (Blimp-1^fL/fL^; Tg^Cre−GNZB^) Blimp-1 conditional KO (CD19, Granzyme B background)	Jeremy Boss laboratory	N/A
Mouse: B6; 129S4-Dnmt3a < tm3.1Enl >	MTA from Riken Institute RBRC037313, Rafi Ahmed laboratory	N/A

**Oligonucleotides**		

5' > 3′ ggtggcctgggatagatcact	Fisher Scientific, this study	BAC ‘203’(*Pdcd1*) forward
5' > 3′ cccgcttccagatcataca	Fisher Scientific, this study	BAC ‘203’(*Pdcd1*) reverse
5' > 3′ gtacgggtgtggaccatcgac	Fisher Scientific, this study	BAC ‘287’(*Sell*) forward
5' > 3′ gccctgggaaagcctcaatac	Fisher Scientific, this study	BAC ‘287’(*Sell*) reverse
5' > 3′ ccacagaagattccgtttgt	Fisher Scientific, this study	BAC ‘353’ (*Ifng*) forward
Mouse Hybloc DNA	Applied Genetic laboratories	Cat# MHB-0.5
5’>3′ gtggaaaaatagctgtagaattg	Integrated DNA technologies	*Prdm1* forward
5’>3′ ctgaggacatgtccccacccactgaaac	Integrated DNA technologies	*Prdm1* reverse

**Recombinant DNA**		

RP23-203H16 (*Pdcd1*) BAC, mouse chromosome 1	BACPAC Resources Online	https://bacpacresources.org
RP24-287N8 (*Sell*) BAC, mouse chromosome 1	BACPAC Resources Online	https://bacpacresources.org
RP23-121J20 (*Cd4*) BAC, mouse chromosome 6	BACPAC Resources Online	https://bacpacresources.org
RP23-139M18 (*Cd8*) BAC, mouse chromosome 6	BACPAC Resources Online	https://bacpacresources.org
R23-353P23 (*Ifng*) BAC, mouse chromosome 10	BACPAC Resources Online	https://bacpacresources.org
mouse *Cot-1* DNA	Invitrogen	Cat # 18840-016
salmon sperm DNA	Ambion	Cat# AM9680

**Software and algorithms**		

FlowJo v.7.6.1 and v.10	TreeStar, FlowJo,LLC	https://www.flowjo.com/
Prism	GraphPad	https://www.graphpad.com/scientific-software/prism/
Systat 13	SYSTAT	https://grafiti.com/systat/
Langsrud.com	N/A	www.langsrud.com/fisher.htm
Zen 2009 and Zen 2 Software	Zeiss	https://www.zeiss.com/microscopy/en/products/software/zeiss-zen.html
ImageJ Software	National Institutes of Health	https://imagej.net/ij/
Ensembl release 46 - Mouse genomes	e!ensembl	https://useast.ensembl.org/Mus_musculus/Info/Index

**Other**		

FACSAriaII BD Biosciences	Emory University	FACSAriaII BD Biosciences
FACSCanto BD Biosciences	Emory University	https://www.bdbiosciences.com/en-us/products/instruments/flow-cytometers/clinical-cell-analyzers/facscanto
Zeiss Laser Scanning Microscope (LSM) 710 Confocal	Zeiss	https://www.zeiss.com/microscopy/en/products/light-microscopes/confocal-microscopes.html

### Resource availability

#### Lead contact

Further information and requests for resources and reagents should be directed to and will be fulfilled by the lead contact, Michael Dustin (michael.dustin@kennedy.ox.ac.uk).

#### Materials availability

Newly generated materials from this study are available upon reasonable request.

#### Data and code availability

• All data supporting the findings of this study are available from the corresponding author upon reasonable request.
• This article does not report any original code.
• Any additional information required to reanalyze the data reported in this article is available from the lead contact upon reasonable request.

### Experimental model and study participant details

#### Mice

C57BL/6 (WT) mice were obtained from the Jackson Laboratory. Transgenic H2-D^b^GP33-41 TCR-tg P14 mice (P14) harboring an engineered TCR recognizing the GP33-41 epitope of LCMV, Blimp-1 conditional KO (*Prdm1*^−/−^) mice (Blimp-1^fL/fL^; Tg^Cre−GNZB^, (CD19, Granzyme B background); described elsewhere^56^), *Dnmt3a*^*−/−*^ (B6; 129S4-Dnmt3a <tm3.1Enl> described elsewhere^64^) were bred and maintained in a closed breeding facility at the Emory Vaccine Center, Emory University. All mice were housed under specific pathogen-free conditions and handled in accordance with the Emory University Institutional Animal Care and Use Committee Guidelines.

### Method details

#### Mouse LCMV infections and generation of antigen-specific CD8^+^ T cells

Single cell suspensions of CD8^+^ splenocytes were purified via MACS CD8^+^ isolation kit II and columns (Miltenyi Biotec) from naive adult (6–8 weeks old) P14 donor mice (Thy1.1^+^). The cells were adoptively transferred intravenously (i.v.) into adult C57BL/6 (Thy1.2^+^) wild-type (WT) mice, *Prdm1*^*−/−*^^56^ (Blimp-1 conditional KO) mice, or *Dnmt3a*^*−/−*^ conditional KO mice, at 6–8 weeks of age, to generate LCMV-specific CD8^+^ T cell chimeras (either 2000 antigen-specific CD8^+^ T cells per mouse, for effector and exhausted cell harvests, or 100,000 cells for memory cell harvests).^86^^,^^87^^,^^88^ 1 day later, chimeric mice were infected with LCMV Armstrong clone 53b (Arm, acute infection) (2 × 10^5^ PFU i.p.) or clone 13 (Cl13, chronic infection) (2 × 10^6^ PFU i.v.), respectively.^10^^,^^85^ Effector Arm and Cl13 CD8^+^ T cells were obtained at 8 days post-infection (dpi). Memory (Arm) and exhausted (Cl13) CD8^+^ T cells were harvested at ≥ 30 dpi from WT mice^9^^,^^15^^,^^42^ and *Dnmt3a*^*−/−*^ mice. For *Pdrm1*^*−/−*^ experiments, effector and exhausted CD8^+^ T cells were harvested as above, at 8 or 28 dpi, respectively. Chimeric antigenic-specific CD8^+^ T cells were FACS-sorted and phenotyped as described below. Naive antigen-specific cells (CD44^lo^) obtained from transgenic P14 mice^89^ were used as an antigen-specific naive control to compare with effector, memory, and exhausted CD8^+^ T cells. For cytokine analysis, *ex vivo* cultures of antigen-specific splenocytes were performed as previously described.^10^ In brief, 1 x 10^6^ splenocytes were cultured for 5 h in a 96-well round bottom plate containing 200 mL of RPMI media supplemented with 10% FBS, L-glutamine (Invitrogen), in the presence of 200 ng/mL of GP33 peptide and Golgiplug (BD Biosciences) as previously described.^43^ Cytokine staining was performed according to manufacturer instructions (Cytofix/Cytoperm kit, BD Biosciences).

#### Flow cytometry

CD8^+^ T cells were stained according to standard procedures (30 min, 4^o^C) with different cocktails of the following fluorescently labeled antibodies (from either BD Biosciences or BioLegend) and tetramer peptides. Chimeric antigenic-specific CD8^+^ T cells were FACS-sorted via fluorescently labeled Thy1.1 (CD90.1), CD44, CD8 antibodies, and either MHC class-I H2-D^b^ tetramers GP33-APC (WT and *Dnmt3a*^*−/−*^ mice), as previously described^56^^,^^64^^,^^90^ or GP33-APC/GP276-APC/NP396-APC (*Prdm1*^*−/−*^ mice).^56^ Used for surface staining were tetramers GP33-APC or GP276-APC/NP396-APC plus anti-mouse antibodies against CD8, CD44, Thy1.1, PD-1 (clone J43), PD-1 (clone 29F.1A12), CD62L (Clone MEL-14), KLRG1, CD27 (TNFR), CD127 (IL-7Rα), CD25 (IL-2), 2B4, CD69, as well as rat anti-mouse IGg2a and rat IgG2bκ isotype controls. Used for intracellular staining were anti-mouse antibodies against IFNγ, TNFα, IL-2, Granzyme B. Cells were sorted on a FACSAriaII (BD Biosciences) and were acquired using a digital flow cytometer FACSCanto (BD Biosciences). All flow cytometric data were analyzed using FlowJo 7.6 and 10 software (TreeStar).

#### Quantitative real-time PCR analysis of mRNA

Total RNA was isolated from purified antigen-specific CD8^+^ T cells from naive mice and mice infected with LCMV Arm and Cl13. RNA was extracted from cells with the RNAeasy kit (QIAGEN) according to manufacturer’s instructions. Quantitative real-time PCR of *Pdcd1* and *Sell* transcripts was performed with primers as previously described.^43^^,^^70^^,^^91^ Transcript expression values were normalized to 18S ribosomal RNA (Applied Biosystems). The DNA primer sequences for *Prdm1* recombination in WT and *Prdm1*^*−/−*^ mice were 5’→3′ Forward: gtggaaaaatagctgtagaattg, Reverse: ctgaggacatgtccccacccactgaaac. Triplicate experiments were analyzed with GraphPad Prism. Statistically significant different transcript expression was assessed by a two-tailed unpaired Student’s t test.

#### Genomic methylation analysis

DNA was isolated (QIAGEN) from LCMV-specific CD8^+^ T cells that were FACS-purified to >95%. Bisulfite-induced deamination of unmethylated cytosines and sequencing of target genomic regions of *Pdcd1* and *Sell* loci were used to measure the allelic frequency of methylated cytosines.^92^ Bisulfite modification was performed with the EZ DNA methylation kit (Zymo Research). The bisulfite-modified DNA was PCR-amplified with locus-specific primers, as previously described.^43^^,^^46^ PCR amplicons, cloned into the pGEM-T TA cloning vector (Promega), were transformed into XL10-Gold ultracompetent bacteria (Stratagene). Individual bacterial colonies were grown overnight, the cloning vectors purified, and the respective *Pdcd1* or *Sell* genomic inserts sequenced. Statistical differences in CpG site methylation were determined using a two-tailed unpaired Student’s t test comparing individual CpG sites from DNA of the described cell populations (GraphPad Prism).

#### BAC probe design for 3D-DNA-FISH

BAC DNA sequences were designed using Ensembl release 46 software based on *Mus musculus* genomic Contig maps for the following loci: *Pdcd1*, *Sell*, both on mouse chromosome 1; and *Ifng*, mouse chromosome 10. The identified and selected BACs, RP23-203H16 (*Pdcd1*), RP24-287N8 (*Sell*), and R23-353P23 (*Ifng*) were then obtained from stock DH10 *E. coli* clones (BACPAC Resources Online). The respective DH10 *E. Coli.* clones derived from libraries constructed in BAC vectors pBACe3.6 or pTARBAC2 (BACPAC Resources Online) were grown in 2xYT medium in 12.5 μg/mL chloramphenicol and BAC DNA purified with HiPure Plasmid DNA purification kit (Invitrogen) or Plasmid Maxiprep DNA kit (QIAGEN). The following oligonucleotide primer pairs (Fisher Scientific) were designed to amplify BAC DNAs 5’→3’: BAC ‘203’(*Pdcd1*) forward ggtggcctgggatagatcact, reverse cccgcttccagatcataca; BAC ‘287’(*Sell*) forward gtacgggtgtggaccatcgac, reverse gccctgggaaagcctcaatac; BAC ‘353’ (*Ifng*) forward ccacagaagattccgtttgt, reverse tggcctttgctgttgggtta.

#### Probe labeling for 3D-DNA-FISH

The following BAC probes were used: RP23-203H16 (*Pdcd1*); RP24-287N8 (*Sell*); R23-353P23 (*Ifng*); and RP23-121J20 (*Cd4*), RP23-139M18 (*Cd8*), both on mouse chromosome 6 (the latter 2, a kind gift from Jane Skok’s laboratory). Probes were directly labeled by nick translation with ChromaTide Alexa Fluor 488-5-dUTP, 546-14-dUTP, 568-5 dUTP or 594-5-dUTP (Invitrogen), or Cy3-dUTP (GE Healthcare), dTTP (GE Healthcare), dNTP (Sigma), DNase I (Roche), DNA polymerase I (NE Biolabs), 0.1 M β-mercaptoethanol (Sigma), in NT buffer, dialyzed in PBS, and combined with 3μg of a 1:1:1 salmon sperm DNA (Ambion), Hybloc DNA (Applied Genentech laboratories) plus mouse *Cot-1* DNA (Invitrogen) to reduce hybridization background. For each coverslip, 1 μg of nick-translation product was precipitated and resuspended in 15 μL of hybridization buffer (50% formamide (Fisher)/10% dextran sulfate (Sigma)/5x Denhardt’s solution (Invitrogen), denatured for 5 min at 95°C, placed on ice 2 min, and pre-annealed for 1h at 37°C before overnight hybridization with cells on coverslips.

#### 3D-DNA FISH and immunofluorescence (immunoFISH)

3D-DNA FISH combined with lamin B immunofluorescence was performed on sorted CD8^+^ T cells from LCMV-infected and naive mice, using variations on previously described methods.^93^ Briefly, cells were washed in PBS, adhered to poly-L-lysine (Sigma) coated to coverslips, and fixed with electron microscopy grade 4% paraformaldehyde/PBS (pH 7–7.4) (EMS) for 10 min at room temperature (RT) in 6-well tissue culture plates. Cells were washed 3 times in PBS, permeabilized for 5 min with 0.4% Triton X-100 (Sigma)/PBS at RT and washed 3 times in PBS. After incubation with 0.1 mg/mL RNaseA (Roche) for 1-2h at 37°C, cells were rinsed 3 times in PBS, denatured with 1.9 M HCl for 30 min at RT, then rinsed again 3 times in ice-cold PBS. Subsequently, cells were hybridized overnight with specific probes at 37°C (coverslips were sealed onto slides with rubber cement). The following day, cells were rinsed in 2x SSC (Ambion) at 37°C, then in 2x SSC at RT, and once in 1x SSC at RT, for 20–30 min each, gentle shaking. Immediately after this, immunofluorescence was performed at RT with samples protected from light. Samples were blocked for 30 min in 4x SSC/3% BSA (EMD Chemicals)/0.05% Tween 20 (Ambion) and incubated for 40 min with a combination of primary antibodies (Abs) against anti-lamin B in blocking solution (1:200 dilution, M20 and C20 anti-lamin Abs, Santa Cruz). After washing 2 times in 4x SSC/0.5% BSA/0.05% Tween 20, cells on coverslips were incubated for 40 min with a secondary donkey-*anti*-goat antibody (Alexa Fluor 647, 1:10000 dilution, Invitrogen), rinsed 3 times in 4x SSC/0.05% Tween 20, gentle rocking, rinsed once in PBS and mounted on slides in ProLong Gold/DAPI buffer (Invitrogen).

#### Confocal microscopy and analysis

3D images were acquired by confocal microscopy on an inverted LSM 710 Zeiss microscope equipped with a spectral detector and employing a Zeiss Plan-Apochromat 63x/1.40 oil objective. The following excitation wavelengths, laser sources, and detection spectra were used: 405 nm/diode laser/418–480nm; 488 nm/argon laser/499–562nm; 543nm/argon laser/564–678nm; 633nm/argon laser/642–720nm. Optical sections separated by 0.3 μm were collected and stacks were analyzed using Zeiss Zen 2009 and Zen 2 software. Alleles were defined as associated with lamin B when BAC signals were adjacent or overlapping with the lamin B signal (no pixel in between the edges of the BAC and lamin B signals). For statistical analysis, a two-tailed Fisher’s exact test was used to analyze the significance of allelic associations with lamin B. The statistical test was applied to combined data from repeated experiments. Sample sizes were in principle, 100–200 cells per experiment, and experiments were repeated at least 2–3 times. Data for individual experiments showed low variation between repeats. In all statistical tests, *p* values ≤0.05 were taken to be significant (0.01 < *p* ≤ 0.05 significant ^∗^; 0.01 < *p* ≤ 0.001 very significant ^∗∗^; *p* ≤ 1.00e-3 highly significant ^∗∗∗^). Statistical significance was calculated across all groups (including ‘none’, ‘monoallelic’, and ‘biallelic’). Graphs and *p* values combine 2–3 independent and representative experiments. See Tables S1–S11 for raw data.

#### Chromatin immunoprecipitation experiments

GP33-specific P14 CD8^+^ T cells were sorted by MACS from the spleens of naive P14 mice. 10,000 cells were adoptively transferred into naive C57BL/6 mice 1 day prior to infection. Adoptively transferred mice were then infected with 2 × 10^5^ PFU LCMV Arm via intraperitoneal (i.p.) injection, or 2 × 10^6^ PFU LCMV Cl13 i.v. (lateral tail vein). At 8 and 28 dpi, antigen-specific P14 cells were sorted from the spleens of LCMV Arm or Cl13-infected mice. For Cl13, 28 dpi infections, spleens from 5 mice were combined for each replicate. Chromatin immunoprecipitation (ChIP) assays were performed as initially described with some modifications.^94^ Cells were cross-linked with 1% formaldehyde for 15 min at room temperature, then subjected to lysis and sonication. Chromatin was incubated with rabbit IgG (Millipore) or anti-Blimp-1 antibody (Rockland) overnight at 4^ο^ C. Protein A beads were added to precipitate the antibody-chromatin complex. Following immunoprecipitation, crosslinks were reversed, DNA purified, and precipitated DNA was subjected to real-time PCR analysis and quantified with a standard curve from sonicated murine genomic DNA. Data were plotted as percentage of input chromatin DNA. Experiments were performed with chromatin from three independent experiments. Statistical analysis was performed using two-tailed unpaired Student’s t test *p* values ns, no significance (*p* > 5.00e-2); ^∗^, significant (5.00e-2 > *p* > 1.00e-2); ^∗∗^, very significant (1.00e-2 > *p* > 1.00e-3); ^∗∗∗^, highly significant (*p* < 1.00e-3) (GraphPad Prism).

### Quantification and statistical analysis

The statistical details of experiments can be found in the ‘Method details’ separate sections, and in the figure legends (including sample sizes and *p* values). For sample size, *n* = the number of mice, as specified. Data analysis was performed using GraphPad Prism, Systat 13, and/or www.langsrud.com/fisher.htm.

## Supplemental information

Document S1. Figures S1–S4 and Tables S1–S11

Document S2. Article plus supplemental information

## References

[1] La Gruta N.L., Kedzierska K., Stambas J., Doherty P.C. (2007). A question of self-preservation: immunopathology in influenza virus infection. Immunol. Cell Biol..

[2] Agata Y., Kawasaki A., Nishimura H., Ishida Y., Tsubata T., Yagita H., Honjo T. (1996). Expression of the PD-1 antigen on the surface of stimulated mouse T and B lymphocytes. Int. Immunol..

[3] Keir M.E., Butte M.J., Freeman G.J., Sharpe A.H. (2008). PD-1 and its ligands in tolerance and immunity. Annu. Rev. Immunol..

[4] Reddy K.L., Zullo J.M., Bertolino E., Singh H. (2008). Transcriptional repression mediated by repositioning of genes to the nuclear lamina. Nature.

[5] Moskophidis D., Lechner F., Pircher H., Zinkernagel R.M. (1993). Virus persistence in acutely infected immunocompetent mice by exhaustion of antiviral cytotoxic effector T cells. Nature.

[6] Wherry E.J. (2011). T cell exhaustion. Nat. Immunol..

[7] Iwai Y., Ishida M., Tanaka Y., Okazaki T., Honjo T., Minato N. (2002). Involvement of PD-L1 on tumor cells in the escape from host immune system and tumor immunotherapy by PD-L1 blockade. Proc. Natl. Acad. Sci. USA.

[8] Ahmed R., Oldstone M.B. (1988). Organ-specific selection of viral variants during chronic infection. J. Exp. Med..

[9] Zajac A.J., Blattman J.N., Murali-Krishna K., Sourdive D.J., Suresh M., Altman J.D., Ahmed R. (1998). Viral immune evasion due to persistence of activated T cells without effector function. J. Exp. Med..

[10] Wherry E.J., Blattman J.N., Murali-Krishna K., van der Most R., Ahmed R. (2003). Viral persistence alters CD8 T-cell immunodominance and tissue distribution and results in distinct stages of functional impairment. J. Virol..

[11] Akondy R.S., Fitch M., Edupuganti S., Yang S., Kissick H.T., Li K.W., Youngblood B.A., Abdelsamed H.A., McGuire D.J., Cohen K.W. (2017). Origin and differentiation of human memory CD8 T cells after vaccination. Nature.

[12] Day C.L., Kaufmann D.E., Kiepiela P., Brown J.A., Moodley E.S., Reddy S., Mackey E.W., Miller J.D., Leslie A.J., DePierres C. (2006). PD-1 expression on HIV-specific T cells is associated with T-cell exhaustion and disease progression. Nature.

[13] Gallimore A., Glithero A., Godkin A., Tissot A.C., Pluckthun A., Elliott T., Hengartner H., Zinkernagel R. (1998). Induction and exhaustion of lymphocytic choriomeningitis virus-specific cytotoxic T lymphocytes visualized using soluble tetrameric major histocompatibility complex class I-peptide complexes. J. Exp. Med..

[14] Petrovas C., Casazza J.P., Brenchley J.M., Price D.A., Gostick E., Adams W.C., Precopio M.L., Schacker T., Roederer M., Douek D.C., Koup R.A. (2006). PD-1 is a regulator of virus-specific CD8+ T cell survival in HIV infection. J. Exp. Med..

[15] Barber D.L., Wherry E.J., Masopust D., Zhu B., Allison J.P., Sharpe A.H., Freeman G.J., Ahmed R. (2006). Restoring function in exhausted CD8 T cells during chronic viral infection. Nature.

[16] Trautmann L., Janbazian L., Chomont N., Said E.A., Gimmig S., Bessette B., Boulassel M.R., Delwart E., Sepulveda H., Balderas R.S. (2006). Upregulation of PD-1 expression on HIV-specific CD8+ T cells leads to reversible immune dysfunction. Nat. Med..

[17] Blackburn S.D., Shin H., Haining W.N., Zou T., Workman C.J., Polley A., Betts M.R., Freeman G.J., Vignali D.A., Wherry E.J. (2009). Coregulation of CD8+ T cell exhaustion by multiple inhibitory receptors during chronic viral infection. Nat. Immunol..

[18] Zinselmeyer B.H., Heydari S., Sacristan C., Nayak D., Cammer M., Herz J., Cheng X., Davis S.J., Dustin M.L., McGavern D.B. (2013). PD-1 promotes immune exhaustion by inducing antiviral T cell motility paralysis. J. Exp. Med..

[19] Zullo J.M., Demarco I.A., Pique-Regi R., Gaffney D.J., Epstein C.B., Spooner C.J., Luperchio T.R., Bernstein B.E., Pritchard J.K., Reddy K.L., Singh H. (2012). DNA sequence-dependent compartmentalization and silencing of chromatin at the nuclear lamina. Cell.

[20] Guelen L., Pagie L., Brasset E., Meuleman W., Faza M.B., Talhout W., Eussen B.H., de Klein A., Wessels L., de Laat W., van Steensel B. (2008). Domain organization of human chromosomes revealed by mapping of nuclear lamina interactions. Nature.

[21] Peric-Hupkes D., Meuleman W., Pagie L., Bruggeman S.W., Solovei I., Brugman W., Graf S., Flicek P., Kerkhoven R.M., van Lohuizen M. (2010). Molecular maps of the reorganization of genome-nuclear lamina interactions during differentiation. Mol. Cell.

[22] Chen C.K., Blanco M., Jackson C., Aznauryan E., Ollikainen N., Surka C., Chow A., Cerase A., McDonel P., Guttman M. (2016). Xist recruits the X chromosome to the nuclear lamina to enable chromosome-wide silencing. Science.

[23] Andrulis E.D., Neiman A.M., Zappulla D.C., Sternglanz R. (1998). Perinuclear localization of chromatin facilitates transcriptional silencing. Nature.

[24] Meister P., Taddei A. (2013). Building silent compartments at the nuclear periphery: a recurrent theme. Curr. Opin. Genet. Dev..

[25] Meister P., Towbin B.D., Pike B.L., Ponti A., Gasser S.M. (2010). The spatial dynamics of tissue-specific promoters during C. elegans development. Genes Dev..

[26] van Steensel B., Belmont A.S. (2017). Lamina-Associated Domains: Links with Chromosome Architecture, Heterochromatin, and Gene Repression. Cell.

[27] Barrales R.R., Forn M., Georgescu P.R., Sarkadi Z., Braun S. (2016). Control of heterochromatin localization and silencing by the nuclear membrane protein Lem2. Genes Dev..

[28] Kosak S.T., Skok J.A., Medina K.L., Riblet R., Le Beau M.M., Fisher A.G., Singh H. (2002). Subnuclear compartmentalization of immunoglobulin loci during lymphocyte development. Science.

[29] Skok J.A., Gisler R., Novatchkova M., Farmer D., de Laat W., Busslinger M. (2007). Reversible contraction by looping of the Tcra and Tcrb loci in rearranging thymocytes. Nat. Immunol..

[30] Hewitt S.L., Yin B., Ji Y., Chaumeil J., Marszalek K., Tenthorey J., Salvagiotto G., Steinel N., Ramsey L.B., Ghysdael J. (2009). RAG-1 and ATM coordinate monoallelic recombination and nuclear positioning of immunoglobulin loci. Nat. Immunol..

[31] Chaumeil J., Micsinai M., Ntziachristos P., Deriano L., Wang J.M., Ji Y., Nora E.P., Rodesch M.J., Jeddeloh J.A., Aifantis I. (2013). Higher-Order Looping and Nuclear Organization of Tcra Facilitate Targeted RAG Cleavage and Regulated Rearrangement in Recombination Centers. Cell Rep..

[32] Towbin B.D., Meister P., Pike B.L., Gasser S.M. (2010). Repetitive transgenes in C. elegans accumulate heterochromatic marks and are sequestered at the nuclear envelope in a copy-number- and lamin-dependent manner. Cold Spring Harbor Symp. Quant. Biol..

[33] Shevelyov Y.Y., Nurminsky D.I. (2012). The nuclear lamina as a gene-silencing hub. Curr. Issues Mol. Biol..

[34] Kind J., Pagie L., Ortabozkoyun H., Boyle S., de Vries S.S., Janssen H., Amendola M., Nolen L.D., Bickmore W.A., van Steensel B. (2013). Single-cell dynamics of genome-nuclear lamina interactions. Cell.

[35] Yanez-Cuna J.O., van Steensel B. (2017). Genome-nuclear lamina interactions: from cell populations to single cells. Curr. Opin. Genet. Dev..

[36] Pickersgill H., Kalverda B., de Wit E., Talhout W., Fornerod M., van Steensel B. (2006). Characterization of the Drosophila melanogaster genome at the nuclear lamina. Nat. Genet..

[37] Filion G.J., van Bemmel J.G., Braunschweig U., Talhout W., Kind J., Ward L.D., Brugman W., de Castro I.J., Kerkhoven R.M., Bussemaker H.J., van Steensel B. (2010). Systematic protein location mapping reveals five principal chromatin types in Drosophila cells. Cell.

[38] Ragoczy T., Bender M.A., Telling A., Byron R., Groudine M. (2006). The locus control region is required for association of the murine beta-globin locus with engaged transcription factories during erythroid maturation. Genes Dev..

[39] Zink D., Amaral M.D., Englmann A., Lang S., Clarke L.A., Rudolph C., Alt F., Luther K., Braz C., Sadoni N. (2004). Transcription-dependent spatial arrangements of CFTR and adjacent genes in human cell nuclei. J. Cell Biol..

[40] Robson M.I., de Las Heras J.I., Czapiewski R., Sivakumar A., Kerr A.R.W., Schirmer E.C. (2017). Constrained release of lamina-associated enhancers and genes from the nuclear envelope during T-cell activation facilitates their association in chromosome compartments. Genome Res..

[41] Fitzsimmons S.P., Bernstein R.M., Max E.E., Skok J.A., Shapiro M.A. (2007). Dynamic changes in accessibility, nuclear positioning, recombination, and transcription at the Ig kappa locus. J. Immunol..

[42] Wherry E.J., Ha S.J., Kaech S.M., Haining W.N., Sarkar S., Kalia V., Subramaniam S., Blattman J.N., Barber D.L., Ahmed R. (2007). Molecular signature of CD8+ T cell exhaustion during chronic viral infection. Immunity.

[43] Youngblood B., Oestreich K.J., Ha S.J., Duraiswamy J., Akondy R.S., West E.E., Wei Z., Lu P., Austin J.W., Riley J.L. (2011). Chronic virus infection enforces demethylation of the locus that encodes PD-1 in antigen-specific CD8(+) T cells. Immunity.

[44] Wartewig T., Kurgyis Z., Keppler S., Pechloff K., Hameister E., Ollinger R., Maresch R., Buch T., Steiger K., Winter C. (2017). PD-1 is a haploinsufficient suppressor of T cell lymphomagenesis. Nature.

[45] Eckersley-Maslin M.A., Spector D.L. (2014). Random monoallelic expression: regulating gene expression one allele at a time. Trends Genet..

[46] Sarkar S., Kalia V., Haining W.N., Konieczny B.T., Subramaniam S., Ahmed R. (2008). Functional and genomic profiling of effector CD8 T cell subsets with distinct memory fates. J. Exp. Med..

[47] Kaech S.M., Hemby S., Kersh E., Ahmed R. (2002). Molecular and functional profiling of memory CD8 T cell differentiation. Cell.

[48] Youngblood B., Hale J.S., Kissick H.T., Ahn E., Xu X., Wieland A., Araki K., West E.E., Ghoneim H.E., Fan Y. (2017). Effector CD8 T cells dedifferentiate into long-lived memory cells. Nature.

[49] Sellars M., Huh J.R., Day K., Issuree P.D., Galan C., Gobeil S., Absher D., Green M.R., Littman D.R. (2015). Regulation of DNA methylation dictates Cd4 expression during the development of helper and cytotoxic T cell lineages. Nat. Immunol..

[50] Collins A., Hewitt S.L., Chaumeil J., Sellars M., Micsinai M., Allinne J., Parisi F., Nora E.P., Bolland D.J., Corcoran A.E. (2011). RUNX transcription factor-mediated association of Cd4 and Cd8 enables coordinate gene regulation. Immunity.

[51] Kallies A., Xin A., Belz G.T., Nutt S.L. (2009). Blimp-1 transcription factor is required for the differentiation of effector CD8(+) T cells and memory responses. Immunity.

[52] Xin A., Masson F., Liao Y., Preston S., Guan T., Gloury R., Olshansky M., Lin J.X., Li P., Speed T.P. (2016). A molecular threshold for effector CD8(+) T cell differentiation controlled by transcription factors Blimp-1 and T-bet. Nat. Immunol..

[53] Rutishauser R.L., Martins G.A., Kalachikov S., Chandele A., Parish I.A., Meffre E., Jacob J., Calame K., Kaech S.M. (2009). Transcriptional repressor Blimp-1 promotes CD8(+) T cell terminal differentiation and represses the acquisition of central memory T cell properties. Immunity.

[54] Shin H.M., Kapoor V., Guan T., Kaech S.M., Welsh R.M., Berg L.J. (2013). Epigenetic modifications induced by Blimp-1 Regulate CD8⁺ T cell memory progression during acute virus infection. Immunity.

[55] Shin H., Blackburn S.D., Intlekofer A.M., Kao C., Angelosanto J.M., Reiner S.L., Wherry E.J. (2009). A role for the transcriptional repressor Blimp-1 in CD8(+) T cell exhaustion during chronic viral infection. Immunity.

[56] Lu P., Youngblood B.A., Austin J.W., Mohammed A.U., Butler R., Ahmed R., Boss J.M. (2014). Blimp-1 represses CD8 T cell expression of PD-1 using a feed-forward transcriptional circuit during acute viral infection. J. Exp. Med..

[57] Bally A.P.R., Neeld D.K., Lu P., Majumder P., Tang Y., Barwick B.G., Wang Q., Boss J.M. (2020). PD-1 Expression during Acute Infection Is Repressed through an LSD1-Blimp-1 Axis. J. Immunol..

[58] Hewitt S.L., Farmer D., Marszalek K., Cadera E., Liang H.E., Xu Y., Schlissel M.S., Skok J.A. (2008). Association between the Igk and Igh immunoglobulin loci mediated by the 3' Igk enhancer induces 'decontraction' of the Igh locus in pre-B cells. Nat. Immunol..

[59] Mackerness K.J., Cox M.A., Lilly L.M., Weaver C.T., Harrington L.E., Zajac A.J. (2010). Pronounced virus-dependent activation drives exhaustion but sustains IFN-gamma transcript levels. J. Immunol..

[60] Ciucci T., Vacchio M.S., Gao Y., Tomassoni Ardori F., Candia J., Mehta M., Zhao Y., Tran B., Pepper M., Tessarollo L. (2019). The Emergence and Functional Fitness of Memory CD4(+) T Cells Require the Transcription Factor Thpok. Immunity.

[61] Kumaran R.I., Spector D.L. (2008). A genetic locus targeted to the nuclear periphery in living cells maintains its transcriptional competence. J Cell Biol.

[62] Utzschneider D.T., Gabriel S.S., Chisanga D., Gloury R., Gubser P.M., Vasanthakumar A., Shi W., Kallies A. (2020). Early precursor T cells establish and propagate T cell exhaustion in chronic infection. Nat. Immunol..

[63] Ahn E., Youngblood B., Lee J., Lee J., Sarkar S., Ahmed R. (2016). Demethylation of the PD-1 Promoter Is Imprinted during the Effector Phase of CD8 T Cell Exhaustion. J. Virol..

[64] Ghoneim H.E., Fan Y., Moustaki A., Abdelsamed H.A., Dash P., Dogra P., Carter R., Awad W., Neale G., Thomas P.G., Youngblood B. (2017). De Novo Epigenetic Programs Inhibit PD-1 Blockade-Mediated T Cell Rejuvenation. Cell.

[65] Sen D.R., Kaminski J., Barnitz R.A., Kurachi M., Gerdemann U., Yates K.B., Tsao H.W., Godec J., LaFleur M.W., Brown F.D. (2016). The epigenetic landscape of T cell exhaustion. Science.

[66] Pauken K.E., Sammons M.A., Odorizzi P.M., Manne S., Godec J., Khan O., Drake A.M., Chen Z., Sen D.R., Kurachi M. (2016). Epigenetic stability of exhausted T cells limits durability of reinvigoration by PD-1 blockade. Science.

[67] Bengsch B., Ohtani T., Khan O., Setty M., Manne S., O'Brien S., Gherardini P.F., Herati R.S., Huang A.C., Chang K.M. (2018). Epigenomic-Guided Mass Cytometry Profiling Reveals Disease-Specific Features of Exhausted CD8 T Cells. Immunity.

[68] Scharer C.D., Bally A.P., Gandham B., Boss J.M. (2017). Cutting Edge: Chromatin Accessibility Programs CD8 T Cell Memory. J. Immunol..

[69] Lee D.U., Agarwal S., Rao A. (2002). Th2 lineage commitment and efficient IL-4 production involves extended demethylation of the IL-4 gene. Immunity.

[70] Oestreich K.J., Yoon H., Ahmed R., Boss J.M. (2008). NFATc1 regulates PD-1 expression upon T cell activation. J. Immunol..

[71] Bally A.P., Austin J.W., Boss J.M. (2016). Genetic and Epigenetic Regulation of PD-1 Expression. J. Immunol..

[72] Alfei F., Kanev K., Hofmann M., Wu M., Ghoneim H.E., Roelli P., Utzschneider D.T., von Hoesslin M., Cullen J.G., Fan Y. (2019). TOX reinforces the phenotype and longevity of exhausted T cells in chronic viral infection. Nature.

[73] Khan O., Giles J.R., McDonald S., Manne S., Ngiow S.F., Patel K.P., Werner M.T., Huang A.C., Alexander K.A., Wu J.E. (2019). TOX transcriptionally and epigenetically programs CD8(+) T cell exhaustion. Nature.

[74] Scott A.C., Dundar F., Zumbo P., Chandran S.S., Klebanoff C.A., Shakiba M., Trivedi P., Menocal L., Appleby H., Camara S. (2019). TOX is a critical regulator of tumour-specific T cell differentiation. Nature.

[75] Yao C., Sun H.W., Lacey N.E., Ji Y., Moseman E.A., Shih H.Y., Heuston E.F., Kirby M., Anderson S., Cheng J. (2019). Single-cell RNA-seq reveals TOX as a key regulator of CD8(+) T cell persistence in chronic infection. Nat. Immunol..

[76] Ghoneim H.E., Zamora A.E., Thomas P.G., Youngblood B.A. (2016). Cell-Intrinsic Barriers of T Cell-Based Immunotherapy. Trends Mol. Med..

[77] Pauken K.E., Wherry E.J. (2015). Overcoming T cell exhaustion in infection and cancer. Trends Immunol..

[78] Saha A., O'Connor R.S., Thangavelu G., Lovitch S.B., Dandamudi D.B., Wilson C.B., Vincent B.G., Tkachev V., Pawlicki J.M., Furlan S.N. (2016). Programmed death ligand-1 expression on donor T cells drives graft-versus-host disease lethality. J. Clin. Invest..

[79] Topalian S.L., Hodi F.S., Brahmer J.R., Gettinger S.N., Smith D.C., McDermott D.F., Powderly J.D., Carvajal R.D., Sosman J.A., Atkins M.B. (2012). Safety, activity, and immune correlates of anti-PD-1 antibody in cancer. N. Engl. J. Med..

[80] Sharma P., Allison J.P. (2015). Immune checkpoint targeting in cancer therapy: toward combination strategies with curative potential. Cell.

[81] Odorizzi P.M., Pauken K.E., Paley M.A., Sharpe A., Wherry E.J. (2015). Genetic absence of PD-1 promotes accumulation of terminally differentiated exhausted CD8+ T cells. J. Exp. Med..

[82] Abdelsamed H.A., Moustaki A., Fan Y., Dogra P., Ghoneim H.E., Zebley C.C., Triplett B.M., Sekaly R.P., Youngblood B. (2017). Human memory CD8 T cell effector potential is epigenetically preserved during in vivo homeostasis. J. Exp. Med..

[83] Utzschneider D.T., Charmoy M., Chennupati V., Pousse L., Ferreira D.P., Calderon-Copete S., Danilo M., Alfei F., Hofmann M., Wieland D. (2016). T Cell Factor 1-Expressing Memory-like CD8(+) T Cells Sustain the Immune Response to Chronic Viral Infections. Immunity.

[84] Tsui C., Kretschmer L., Rapelius S., Gabriel S.S., Chisanga D., Knopper K., Utzschneider D.T., Nussing S., Liao Y., Mason T. (2022). MYB orchestrates T cell exhaustion and response to checkpoint inhibition. Nature.

[85] Matloubian M., Somasundaram T., Kolhekar S.R., Selvakumar R., Ahmed R. (1990). Genetic basis of viral persistence: single amino acid change in the viral glycoprotein affects ability of lymphocytic choriomeningitis virus to persist in adult mice. J. Exp. Med..

[86] Blattman J.N., Antia R., Sourdive D.J., Wang X., Kaech S.M., Murali-Krishna K., Altman J.D., Ahmed R. (2002). Estimating the precursor frequency of naive antigen-specific CD8 T cells. J. Exp. Med..

[87] Kersh E.N. (2006). Impaired memory CD8 T cell development in the absence of methyl-CpG-binding domain protein 2. J. Immunol..

[88] Kersh E.N., Fitzpatrick D.R., Murali-Krishna K., Shires J., Speck S.H., Boss J.M., Ahmed R. (2006). Rapid demethylation of the IFN-gamma gene occurs in memory but not naive CD8 T cells. J. Immunol..

[89] Pircher H., Burki K., Lang R., Hengartner H., Zinkernagel R.M. (1989). Tolerance induction in double specific T-cell receptor transgenic mice varies with antigen. Nature.

[90] Murali-Krishna K., Altman J.D., Suresh M., Sourdive D.J., Zajac A.J., Miller J.D., Slansky J., Ahmed R. (1998). Counting antigen-specific CD8 T cells: a reevaluation of bystander activation during viral infection. Immunity.

[91] Furukawa Y., Umemoto E., Jang M.H., Tohya K., Miyasaka M., Hirata T. (2008). Identification of novel isoforms of mouse L-selectin with different carboxyl-terminal tails. J. Biol. Chem..

[92] Trinh B.N., Long T.I., Laird P.W. (2001). DNA methylation analysis by MethyLight technology. Methods.

[93] Skok J.A., Brown K.E., Azuara V., Caparros M.L., Baxter J., Takacs K., Dillon N., Gray D., Perry R.P., Merkenschlager M., Fisher A.G. (2001). Nonequivalent nuclear location of immunoglobulin alleles in B lymphocytes. Nat. Immunol..

[94] Beresford G.W., Boss J.M. (2001). CIITA coordinates multiple histone acetylation modifications at the HLA-DRA promoter. Nat. Immunol..

