# Supplementary material for: Chronic viral infection alters PD-1 locus subnuclear localization in cytotoxic CD8+ T cells
Source: Cell Rep. 2024 Jul 30;43(8):114547. doi: 10.1016/j.celrep.2024.114547 (PMC11522508; doi:10.1016/j.celrep.2024.114547)
Supplement: Document S1. Figures S1–S4 and Tables S1–S11 [file mmc1.pdf]

Cell Reports, Volume *43*

Supplemental information

Chronic viral infection alters PD-1 locus subnuclear localization in cytotoxic CD8^{+} T cells

Catarina Sacristán, Ben A. Youngblood, Peiyuan Lu, Alexander P.R. Bally, Jean Xiaojin Xu, Katelyn McGary, Susannah L. Hewitt, Jeremy M. Boss, Jane A. Skok, Rafi Ahmed, and Michael L. Dustin

SUPPLEMENTAL INFORMATION

Supplemental Figures

Sacristan et al. Suppl Figure S1.

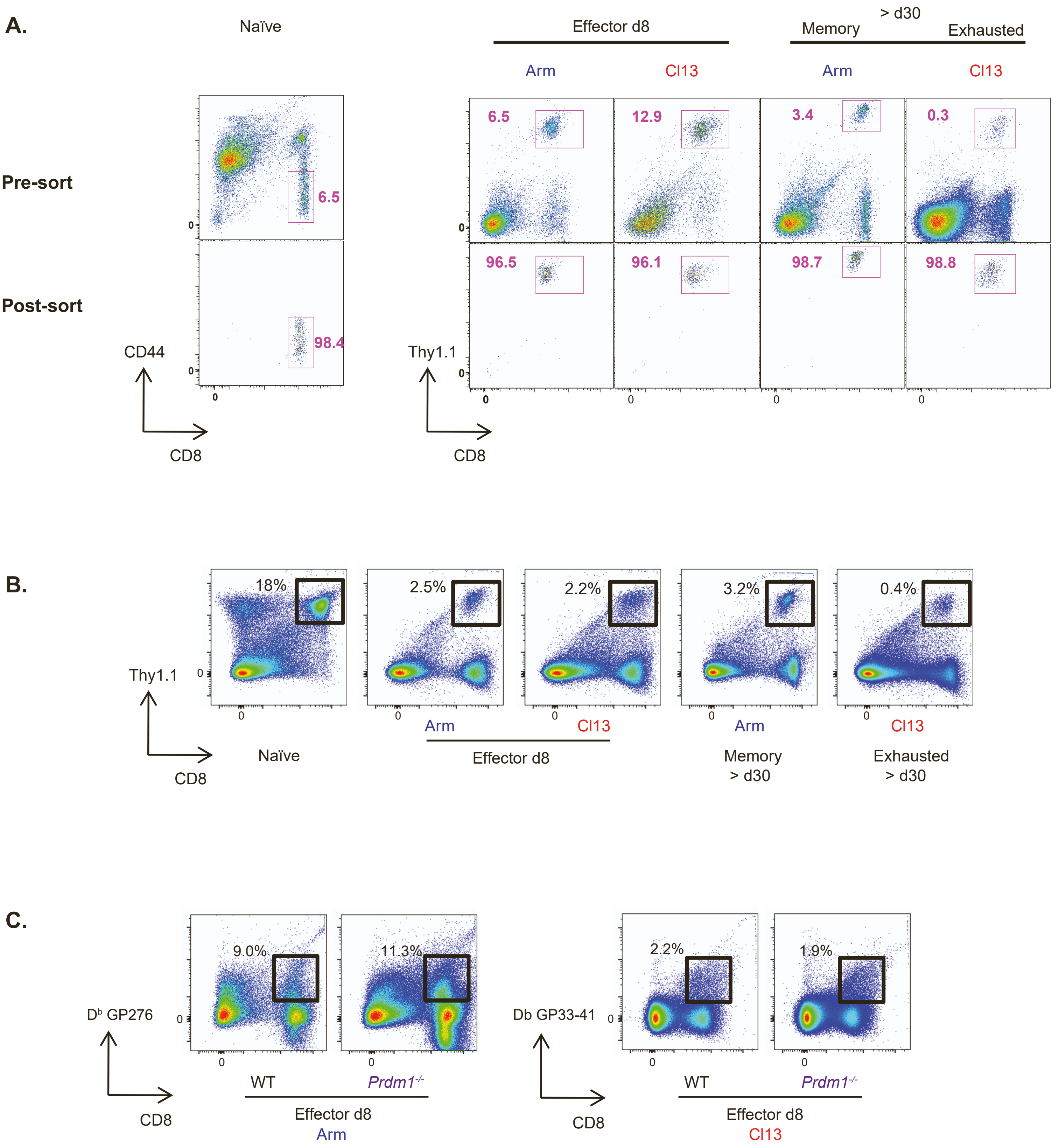

Figure S1. Related to Figures 1-4 and S3. Purification analysis and FACS gating strategies of LCMV-specific CD8$^+$ T cell subsets used in DNA-FISH experiments. CD8$^+$ splenocytes from naïve P14 mice (Thy1.1$^+$) were adoptively transferred into C57BL/6 (Thy1.2$^+$) WT mice to generate LCMV-specific CD8$^+$ T cell chimeras. WT C57BL/6 mice were subsequently infected with either LCMV Armstrong (Arm, *blue*) or LCMV Clone 13 (Cl13, *red*). Antigen-specific CD8$^+$ splenocytes were harvested and purified by FACS using Thy1.1, CD8, and CD44 antibodies. Effector cells were harvested at 8 dpi. Memory and exhausted cells were harvested at > 30 dpi. Naïve antigen-specific cells were obtained from uninfected P14 mice and used as a control to compare against effector, memory, and exhausted CD8$^+$ T cells. (A). Purification analysis of FACS-sorted antigen-specific cells from acutely or chronically infected mice (pre-sort and post-sort) is shown. The number frequencies (%, *pink*) of sorted antigen-specific CD8$^+$ Thy1.1$^+$ cells for naïve, effector, memory, and exhausted T cell subsets are shown. (B-C). Gating strategy of FACS-sorted antigen-specific cells from acutely or chronically infected mice. The number frequencies (%, *black*) of sorted antigen-specific CD8$^+$ Thy1.1$^+$ cells for naïve, effector, memory, and exhausted T cell subsets are shown. (C) Gating strategy of FACS-sorted antigen-specific cells from chronically infected Blimp-1 conditional KO (*Prdm1*$^{-/-}$) and WT C57BL/6 mice is shown. Mice were infected with LCMV Cl13 and splenocytes harvested at 8 dpi. Antigen-specific CD8$^+$ T cells were FACS-purified using an anti-CD8 antibody and a class-I tetramer, specific for the LCMV epitopes D^b GP276 (Arm) or D^b GP33-41 (Cl13). Number frequencies (%, *black*) of sorted antigen-specific CD8$^+$ D^b GP276 or D^b GP33-41$^+$ 8 dpi effector T cells are shown. Data are representative of at least 3 experiments, with n=3 mice per condition, per experiment.

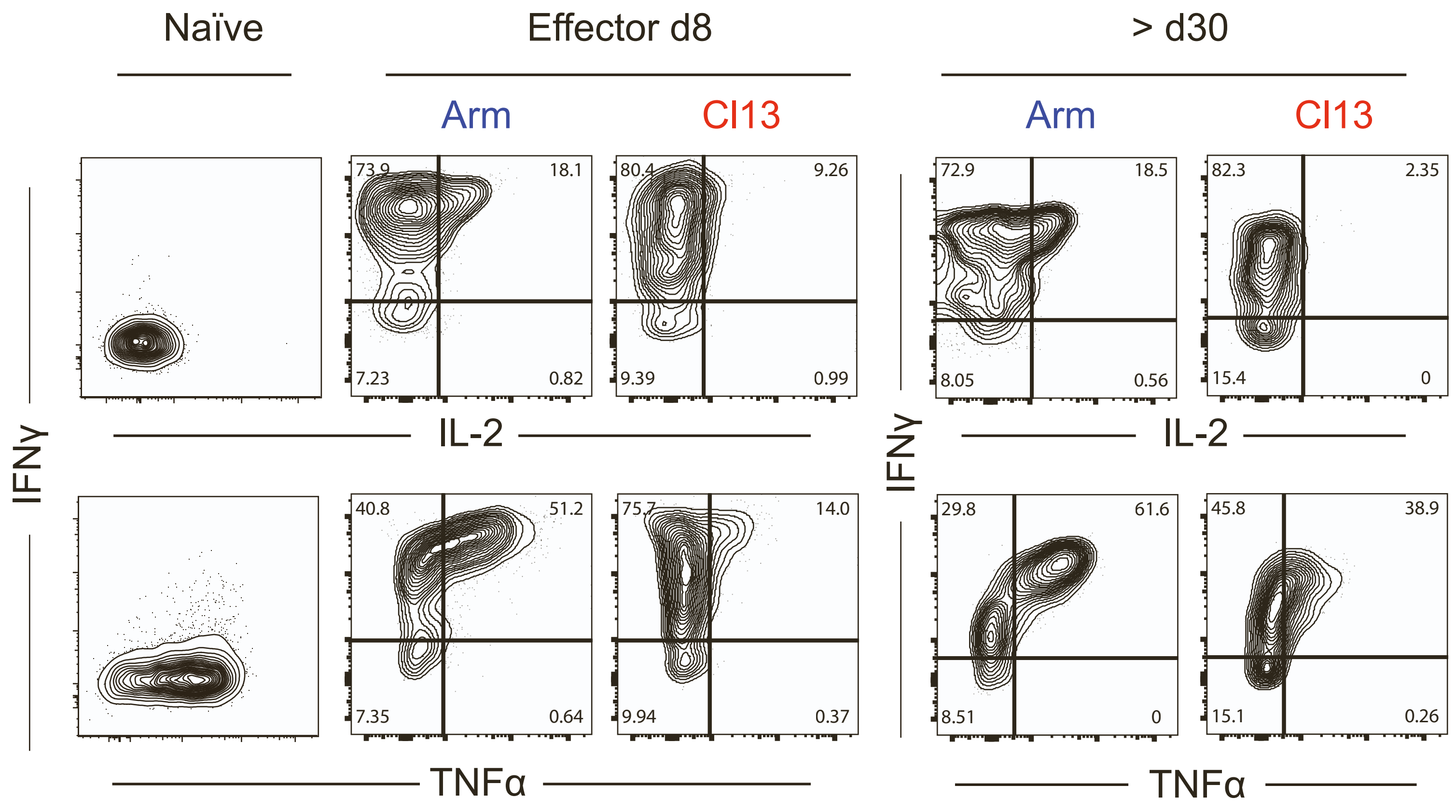
Naïve
Effector d8
> d30
Arm
Cl13
Arm
Cl13
73.9
18.1
7.23
0.82
80.4
9.26
9.39
0.99
72.9
18.5
8.05
0.56
82.3
2.35
15.4
0
IL-2
IL-2
IFNγ
IFNγ
40.8
51.2
7.35
0.64
75.7
14.0
9.94
0.37
29.8
61.6
8.51
0
45.8
38.9
15.1
0.26
TNFα
TNFα

Figure S2. Related to Figures 1-4 and S3. Functional analysis of LCMV-specific CD8$^+$ T cell subsets used in DNA-FISH experiments. Intracellular cytokine FACS analysis of antigen-specific cells from acutely (Arm, *blue*) or chronically (Cl13, *red*) infected mice (see Figure S1) was performed. *Ex vivo* antigen-specific cells at the denoted dpi were cultured in the absence or presence of 200 ng/ml of GP33 peptide and Golgiplug™ (BD Biosciences) for 5h. Shown are the number frequencies of IFN-γ, TNF-α, and IL-2 producing antigen-specific CD8$^+$ Thy1.1$^+$ T cells from the same source as in Figure S1 A, B. The cytokine frequencies were determined following background subtraction of the events from media alone control wells. Data are representative of at least 3 experiments, with n=3 mice per condition, per experiment.

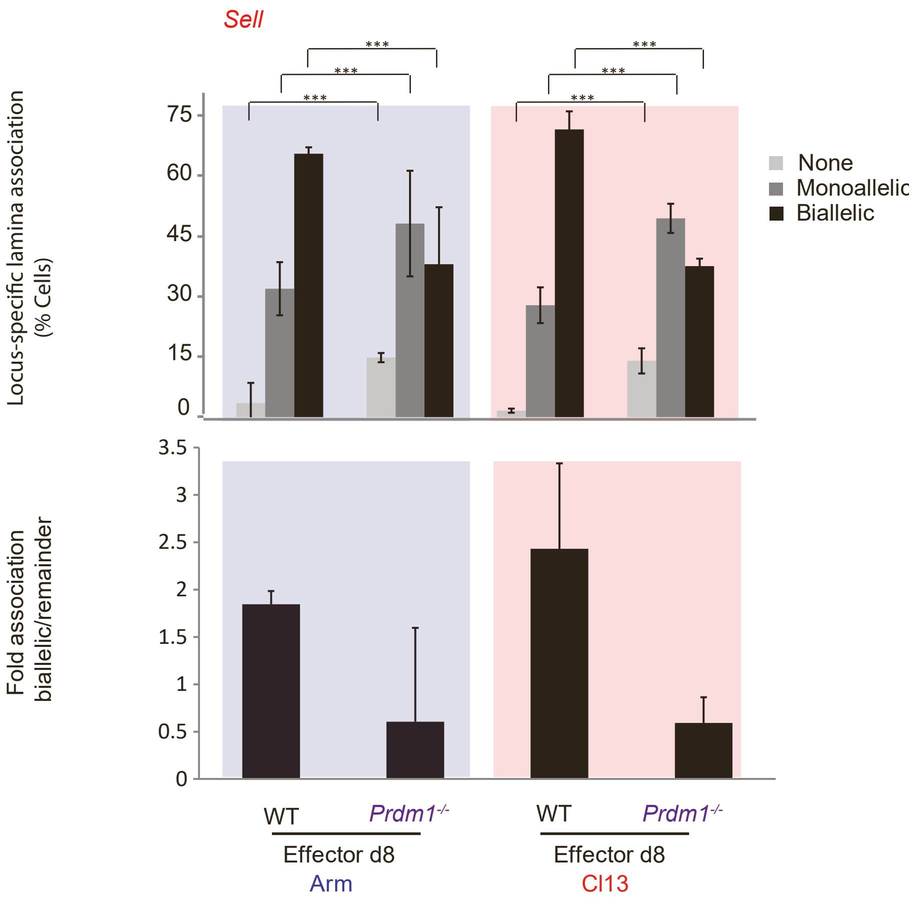
Sell
***
***
***
***
***
***
Locus-specific lamina association (% Cells)
75
60
45
30
15
0
None
Monoalleic
Biallelic
Fold association biallelic/remainder
3.5
3
2.5
2
1.5
1
0.5
0
WT
Prdm1-/-
WT
Prdm1-/-
Effector d8
Arm
Effector d8
Cl13

Figure S3. Related to Figures 4, S1, and S2. Blimp-1 can modulate *Sell* subnuclear localization to lamin in antigen-specific effector CD8$^+$ T cells. LCMV-specific effector CD8$^+$ T cells from acutely from acutely (Arm, *blue*) or chronically infected (Cl13, *red*) Blimp-1-conditional KO (*Prdm1*$^{-/-}$) and WT mice were obtained at 8 dpi and biallelic *Sell* association with lamin B was scored by DNA-immunoFISH. Samples corresponding to Figure 4 are shown. *Top graphs*: The frequencies of cells with *Sell* no association (none), monoallelic, or biallelic locus-specific association with lamin B by DNA-immunoFISH are shown (% Cells). *Bottom graphs*: *Sell* biallelic fold-association with lamina was calculated relative to the remaining conditions (remainder = none + monoallelic). Representative and independent experiments were reproduced at least n=2-3, with n=3 mice per condition, per experiment. The analysis of FISH samples was conducted in at least 100 cells. *P* values (two-tailed Fisher's exact test): ns, no significance (p > 5.00e-2); *, significant (5.00e-2 > *P* > 1.00e-2); **, very significant (1.00e-2 > *P* > 1.00e-3); ***, highly significant (*P* < 1.00e-3). Statistical significance was calculated across all groups (including 'none', 'monoallelic', and 'biallelic' (Tables S8, S9). Graphs and *P* values combine two-three independent and representative experiments. Errors bars represent values +/- standard deviation.

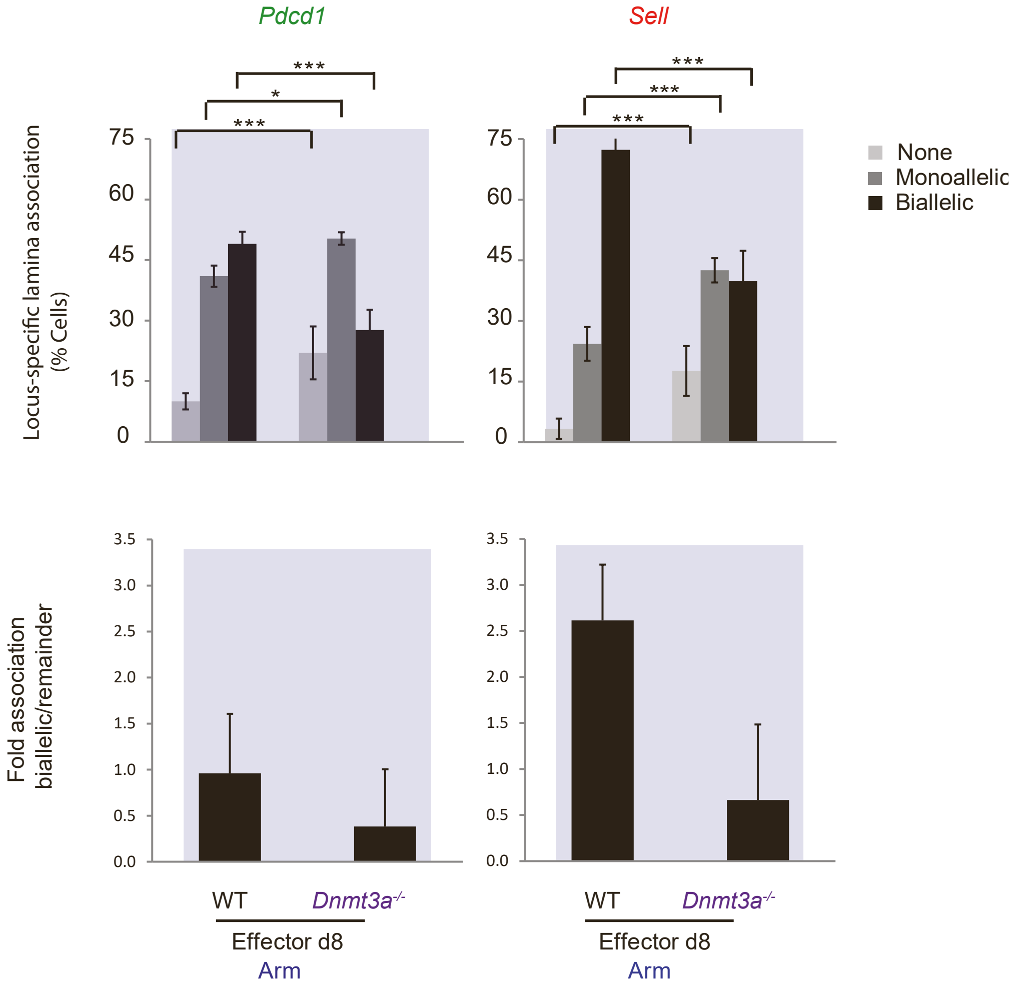
Pdcd1
Sell
***
*
***
***
***
***
Locus-specific lamina association (% Cells)
75
60
45
30
15
0
None
Monoallelic
Biallelic
Fold association biallelic/remainder
3.5
3.0
2.5
2.0
1.5
1.0
0.5
0.0
WT
Dnmt3a-/-
Effector d8
Arm

Figure S4. Related to Figures 1 and 4. Dnmt3a can modulate *Pdcd1* and *Sell* subnuclear localization to lamin in antigen-specific effector CD8⁺ T cells. Dnmt3a conditional KO mice *Dnmt3a$^{-/-}$* that were previously generated by crossing floxed Dnmt3a mice with mice expressing a Granzyme b–driven recombinase transgene (previously described)[1] were infected with Arm LCMV as described (see methods section). The mice were bred and maintained in a closed breeding facility at the Emory Vaccine Center, Emory University. All mice were housed under specific pathogen-free conditions and handled in accordance with the Emory University Institutional Animal Care and Use Committee Guidelines. LCMV-specific effector $CD8^+$ T cells from acutely (Arm, *blue*) *Dnmt3a* $^{-/-}$ and WT mice were obtained at 8 dpi and biallelic *Pdcd1* or *Sell* association with lamin B was scored by DNA-immunoFISH. *Top graphs*: The frequencies of cells with *Pdcd1* (*left*) or *Sell* (right), monoallelic or biallelic locus-specific association with lamin B by DNA-immunoFISH are shown (% Cells). *Bottom graphs*: The *Pdcd1* (*left)* or *Sell* (*right*) biallelic fold-association with lamina was calculated relative to the remaining conditions (none + monoallelic). Representative and independent experiments were reproduced at least n=2-3, with n=3 mice per condition, per experiment. The analysis of FISH samples was conducted in at least 100 cells. *P* values (two-tailed Fisher's exact test): ns, no significance (p > 5.00e-2); *, significant (5.00e-2 > *P* > 1.00e-2); **, very significant (1.00e-2 > *P* > 1.00e-3); ***, highly significant (*P* < 1.00e-3). Statistical significance was calculated across all groups (including 'none', 'monoallelic', and 'biallelic' (Tables S10, S11). Graphs and *P* values combine two-three independent and representative experiments. Errors bars represent values +/- standard deviation. Sort, gating strategy, and intracellular staining performed as in Figures S1 and S2 (data not shown).

1. Ghoneim, H.E., Fan, Y., Moustaki, A., Abdelsamed, H.A., Dash, P., Dogra, P., Carter, R., Awad, W., Neale, G., Thomas, P.G., and Youngblood, B. (2017). De Novo Epigenetic Programs Inhibit PD-1 Blockade-Mediated T Cell Rejuvenation. Cell *170,* 142-157 e119. 10.1016/j.cell.2017.06.007.

Supplemental Video

Video S1. Related to Figure 1C. Biallelic positioning of *Cd4* and *Cd8* loci in an LCMV-specific CTL. The video depicts a 3D-rendering of representative DNA-immunoFISH confocal microscopy Z-section reconstruction of an LCMV-specific mouse memory $CD8^+$ T cell (from Arm LCMV infected mice) showing, in this example, *Cd4 (red)* and *Cd8 (green)* pairs of alleles relative to lamin B (*cyan*).

Supplemental Tables

Statistical analyses were performed using a two-tailed exact Fisher's test.

$P > 0.05$ Not significant =ns
$0.01 < P \leq 0.05$ Significant = *
$0.01 < P \leq 0.001$ Very significant = **
$P \leq 1.00e\text{-}3$ Highly significant = ***

Abbreviations:

Cl13 = Clone 13

Arm = Armstrong

SD = Standard deviation

Table S1. Related to Figure 1. Frequency of ***Pdcd1*** association to nuclear lamina in LCMV-specific CD8$^+$ T cells during acute and chronic infection - DNA-FISH.

A. Overall ***Pdcd1*** association to nuclear lamina (number of alleles).

Pdcd1 CD8$^+$ T cell subset			Associated alleles numbers	%	No association numbers	%	sample size (alleles)	SD
	**Naïve**	Exp 1	289	65.7	151	34.3	440	
		Exp 2	116	56.9	88	43.1	204	
		Exp 3	117	58.5	83	41.5	200	
		Exp 4	144	72.0	56	28.0	200	
		Exp 5	144	72.0	56	28.0	200	
		**Total**	**810**	**65.0**	**434**	**35.0**	**1244**	**7.2**
Day 8	**Effector Arm**	Exp 1	281	70.2	119	29.8	400	
		Exp 2	140	70.0	60	30.0	200	
		Exp 3	141	70.5	59	29.5	200	
		**Total**	**562**	**70.3**	**238**	**29.7**	**800**	**0.3**
	**Effector Cl13**	Exp 1	290	64.4	160	35.6	450	
		Exp 2	143	68.7	65	31.2	208	
		Exp3	135	67.5	65	32.5	200	
		Exp 4	146	70.2	62	29.8	208	
		**Total**	**714**	**67.7**	**352**	**32.3**	**1066**	**2.5**
> Day 30	**Memory Arm**	Exp 1	267	66.7	133	33.3	400	
		Exp 2	125	62.5	75	37.5	200	
		Exp 3	120	61.2	76	38.8	196	
		Exp 4	137	58.0	99	42.0	236	
		Exp 5	139	69.5	61	30.5	200	
		Exp 6	113	57.0	86	43.0	200	
		**Total**	**301**	**62.5**	**530**	**37.5**	**1432**	**4.9**
	**Exhausted Cl13**	Exp 1	163	40.7	237	59.3	400	
		Exp 2	125	39.3	75	60.7	200	
		Exp 3	172	44.6	214	55.4	386	
		Exp 4	79	39.5	121	60.5	200	
		**Total**	**498**	**41.0**	**702**	**59.0**	**1188**	**2.5**

Statistical analysis (Fisher's 2-tail exact test - combined frequency)	*P*-value	Level
**Naïve vs Effector d8 Arm**	0.0159	*
**Naïve vs Effector d8 Cl13**	0.355	**ns**
**Naïve vs Memory**	0.258	**ns**
**Naïve vs Exhausted**	8.46e-32	***
**Effector d8 Arm vs Effector d8 Cl13**	0.145	**ns**
**Effector d8 Arm vs Exhausted**	4.50e-37	***
**Effector d8 Cl13 vs Exhausted**	4.10e-34	***
**Effector d8 Arm vs Memory**	4.948e-4	***
**Effector d8 Cl13 vs Memory**	0.0382	*
**Memory vs Exhausted**	4.43e-28	***

B. Monoallelic and biallelic ***Pdcd1*** association to nuclear lamina (number of cells).

Pdcd1 CD8+ T cell subset			Monoallelic association numbers	%	Biallelic association numbers	%	No association numbers	%	sample size (cells)
	**Naïve**	Exp 1	113	51.4	88	40.0	19	8.6	220
		Exp 2	46	45.1	35	34.3	21	20.6	102
		Exp 3	55	55.0	31	31.0	14	14.0	100
		Exp 4	44	44.0	50	50.0	6	6.0	100
		Exp 5	40	40.0	52	52.0	8	8.0	100
		**Total**	**298**	**47.1**	**256**	**41.5**	**68**	**11.4**	**622**
Day 8	**Effector Arm**	Exp 1	67	33.5	107	53.5	26	13.0	200
		Exp 2	44	44.0	48	48.0	8	8.0	100
		Exp 3	43	43.0	49	49.0	8	8.0	100
		**Total**	**154**	**40.2**	**204**	**50.2**	**42**	**9.7**	**400**
	**Effector Cl13**	Exp 1	112	49.8	89	39.6	24	10.7	225
		Exp 2	45	43.3	49	47.1	10	9.6	104
		Exp3	45	45.0	45	45.0	10	10.0	100
		Exp 4	44	42.3	51	49.0	9	8.7	104
		**Total**	**246**	**45.1**	**234**	**45.2**	**53**	**9.7**	**533**
>Day 30	**Memory Arm**	Exp 1	83	41.5	92	46.0	25	12.5	200
		Exp 2	43	43.0	41	41.0	16	16.0	100
		Exp 3	52	53.1	34	34.7	12	12.2	98
		Exp 4	66	55.9	38	32.2	19	16.1	118
		Exp 5	37	37.0	51	51.0	12	12.0	100
		Exp 6	65	65.0	24	24.0	11	11.0	100
		**Total**	**346**	**49.2**	**280**	**38.1**	**95**	**13.3**	**716**
	**Exhausted Cl13**	Exp 1	101	50.5	31	15.5	68	34.0	200
		Exp 2	52	48.6	16	15.0	39	36.4	107
		Exp 3	96	49.7	38	19.7	59	30.6	193
		Exp 4	47	47.0	16	16.0	37	37.0	100
		**Total**	**296**	**49.0**	**101**	**16.5**	**203**	**34.5**	**600**

Pdcd1 CD8+ T cell subset		Monoallelic association SD	Biallelic association SD	No association SD
	**Naïve**	6.0	9.3	5.9
Day 8	**Effector Arm**	5.8	2.9	2.9
	**Effector Cl13**	3.3	4.1	0.8
> Day 30	**Memory Arm**	10.5	9.8	2.2
	**Exhausted Cl13**	1.5	2.1	2.9

Statistical analysis (Fisher's 2-tail exact test - combined frequency)	*P*-value	Level	*P*-value	Level	*P*-value	Level
**Naïve vs Effector d8 Arm**	3.665e-3	**	2.447e-3	**	0.918	**ns**
**Naïve vs Effector d8 Cl13**	0.555	**ns**	0.370	**ns**	0.630	**ns**
**Naïve vs Memory**	1.000	**ns**	0.402	**ns**	0.241	**ns**
**Naïve vs Exhausted**	0.647	**ns**	4.625e-21	***	1.820e-22	***
**Effector d8 Arm vs Effector d8 Cl13**	2.30e-2	*	0.034	*	0.827	**ns**
**Effector d8 Arm vs Exhausted**	7.50e-4	***	2.289e-30	***	3.320e-18	***
**Effector d8 Cl13 vs Exhausted**	0.311	**ns**	1.790e-23	***	1.077e-22	***
**Effector d8 Arm vs Memory**	2.58e-3	**	9.406e-5	***	0.216	**ns**
**Effector d8 Cl13 vs Memory**	0.529	**ns**	0.072	**ns**	0.092	**ns**

**Memory vs Exhausted**	0.658	**ns**	4.751e-19	***	4.716e-19	***

Table S2. Related to Figure 1. Frequency of ***Sell*** association to nuclear lamina in LCMV-specific CD8$^+$ T cells during acute and chronic infection - DNA-FISH.

A. Overall ***Sell*** association to nuclear lamina (number of alleles).

Sell CD8$^+$ T cell subset			Associated alleles numbers	Associated alleles %	No association numbers	No association %	sample size (alleles)	SD
	**Naïve**	Exp 1	50	24.0	158	76.0	208	
		Exp 2	54	24.8	164	75.2	218	
		Exp 3	59	29.5	141	70.5	200	
		Exp 4	59	29.5	141	70.5	200	
		Exp 5	58	29.0	142	71.0	200	
		**Total**	**280**	**27.4**	**746**	**72.6**	**1026**	**2.7**
Day 8	**Effector Arm**	Exp 1	173	79.4	45	20.6	218	
		Exp 2	171	84.7	31	15.3	202	
		Exp 3	175	87.5	25	12.5	200	
		**Total**	**519**	**83.9**	**101**	**16.1**	**620**	**4.1**
	**Effector Cl13**	Exp 1	177	85.1	31	14.9	208	
		Exp 2	189	86.7	29	13.3	218	
		Exp3	170	85.0	30	15.0	200	
		Exp 4	171	82.2	37	17.8	208	
		**Total**	**707**	**84.8**	**127**	**15.3**	**834**	**1.9**
> Day 30	**Memory Arm**	Exp 1	168	52.8	150	47.2	318	
		Exp 2	160	76.9	48	23.1	208	
		Exp 3	117	58.5	83	41.5	200	
		Exp 4	93	46.0	109	54.0	202	
		Exp 5	121	60.5	79	39.5	200	
		Exp 6	81	40.5	119	60.5	200	
		**Total**	**740**	**55.9**	**588**	**44.3**	**1328**	**12.8**
	**Exhausted Cl13**	Exp 1	268	80.7	64	19.3	332	
		Exp 2	155	76.7	47	23.3	202	
		Exp 3	180	79.0	48	21.0	228	
		Exp 4	168	84.0	32	16.0	200	
		**Total**	**771**	**80.1**	**191**	**19.9**	**962**	**3.1**

Statistical analysis (Fisher's 2-tail exact test - combined frequency)	*P*-value	Level
**Naïve vs Effector d8 Arm**	3.646e-116	***
**Naïve vs Effector d8 Cl13**	1.845e-135	***
**Naïve vs Memory**	3.384e-44	***
**Naïve vs Exhausted**	1.507e-129	***
**Effector d8 Arm vs Effector d8 Cl13**	0.610	**ns**
**Effector d8 Arm vs Exhausted**	0.084	**ns**
**Effector d8 Cl13 vs Exhausted**	0.011	*
**Effector d8 Arm vs Memory**	9.966e-36	***
**Effector d8 Cl13 vs Memory**	3.275e-47	***
**Memory vs Exhausted**	2.959e-35	***

B. Monoallelic and biallelic ***Sell*** association to nuclear lamina (number of cells).

Sell CD8$^+$ T cell subset			Monoallelic association numbers	%	Biallelic association numbers	%	No association numbers	%	sample size (cells)
	Naïve	Exp 1	30	28.8	10	9.6	64	61.5	104
		Exp 2	34	31.2	10	9.2	65	59.6	109
		Exp 3	47	47.0	6	6.0	47	47.0	100
		Exp 4	41	41.0	9	9.0	50	50.0	100
		Exp 5	40	40.0	9	9.0	51	51.0	100
		**Total**	**192**	**37.6**	**44**	**8.6**	**277**	**53.8**	**513**
Day 8	Effector Arm	Exp 1	29	26.6	72	66.1	8	7.3	109
		Exp 2	25	24.8	73	72.3	3	3.0	101
		Exp 3	23	23.0	76	76.0	1	1.0	100
		**Total**	**77**	**24.8**	**221**	**71.4**	**12**	**3.8**	**310**
	Effector Cl13	Exp 1	31	29.8	73	70.2	0	0.0	104
		Exp 2	25	22.9	82	75.2	2	1.8	109
		Exp3	28	28.0	71	71.0	1	1.0	100
		Exp 4	33	31.7	69	66.3	2	1.9	104
		**Total**	**84**	**26.9**	**226**	**72.1**	**3**	**0.9**	**313**
>Day 30	Memory Arm	Exp 1	68	42.8	50	31.4	41	25.8	159
		Exp 2	30	28.8	65	62.5	9	8.7	104
		Exp 3	53	53.0	32	32.0	15	15.0	100
		Exp 4	47	46.5	23	22.8	31	30.7	101
		Exp 5	45	45.0	38	38.0	17	17.0	100
		Exp 6	53	53.0	14	14.0	33	33.0	100
		**Total**	**296**	**44.9**	**222**	**33.5**	**146**	**21.7**	**664**
	Exhausted Cl13	Exp 1	46	27.7	111	66.9	9	5.4	166
		Exp 2	33	32.7	61	60.4	7	6.9	101
		Exp 3	42	36.8	69	60.5	3	2.6	114
		Exp 4	26	26.0	71	71.0	3	3.0	100
		**Total**	**147**	**30.8**	**312**	**64.7**	**22**	**4.5**	**481**

Sell CD8$^+$ T cell subset		Monoallelic association SD	Biallelic association SD	No association SD
	Naïve	7.5	1.5	6.4
Day 8	Effector Arm	1.8	5.0	3.2
	Effector Cl13	3.8	3.6	0.9
> Day 30	Memory Arm	8.9	16.5	9.6
	Exhausted Cl13	4.9	5.2	2.0

Statistical analysis (Fisher's 2-tail exact test - combined frequency)	*P*-value	Level	*P*-value	Level	*P*-value	Level
Naïve vs Effector d8 Arm	2.247e-4	***	2.301e-80	***	2.013e-57	***
Naïve vs Effector d8 Cl13	2.630e-3	**	5.559e-92	***	1.531e-83	***
Naïve vs Memory	0.014	*	8.876e-26	***	6.873e-30	***
Naïve vs Exhausted	0.023	*	1.665e-82	***	4.344e-73	***
Effector d8 Arm vs Effector d8 Cl13	0.352	**ns**	0.934	**ns**	0.024	**ns**
Effector d8 Arm vs Exhausted	0.089	**ns**	0.063	**ns**	0.721	**ns**
Effector d8 Cl13 vs Exhausted	0.420	**ns**	0.063	**ns**	2.95e-3	**
Effector d8 Arm vs Memory	2.353e-9	***	1.146e-28	***	7.077e-15	***
Effector d8 Cl13 vs Memory	4.380e-8	***	1.969e-33	***	1.460e-27	***

**Memory vs Exhausted**	1.571e-6	***	5.756e-26	***	3.375e-18	***

Table S3. Related to Figure 1C. Frequency of control ***Cd4* and *Cd8*** association to nuclear lamina in LCMV-specific CD8$^+$ T cells during acute and chronic infection - DNA-FISH.

A. Overall ***Cd4*** association to nuclear lamina (number of alleles).

Cd4 CD8$^+$ T cell subset			Associated alleles numbers	%	No association numbers	%	sample size (alleles)	SD
	**Naïve**	Exp 1	47	23.5	153	76.5	200	
		Exp 2	53	22.8	147	77.2	200	
		**Total**	**100**	**25.0**	**300**	**75.0**	**400**	**2.1**
> Day 30	**Memory Arm**	Exp 1	52	26.0	148	74.0	200	
		Exp 2	55	27.5	145	72.5	200	
		**Total**	**107**	**26.8**	**293**	**73.3**	**400**	**1.1**
	**Exhausted Cl13**	Exp 1	46	22.8	156	77.2	202	
		Exp 2	64	32	136	68	200	
		**Total**	**110**	**27.4**	**292**	**72.6**	**402**	**6.5**

Statistical analysis (Fisher's 2-tail exact test - combined frequency)	*P*-value	Level
**Naïve vs Memory**	0.628	**ns**
**Naïve vs Exhausted**	0.470	**ns**
**Memory vs Exhausted**	0.874	**ns**

B. Monoallelic and biallelic ***Cd4*** association to nuclear lamina (number of cells).

Cd4 CD8$^+$ T cell subset			Monoallelic association numbers	%	Biallelic association numbers	%	No association numbers	%	sample size (cells)
	**Naïve**	Exp 1	39	39.0	4	4.0	57	57.0	100
		Exp 2	37	37.0	8	8.0	55	55.0	100
		**Total**	**76**	**38.0**	**12**	**6.0**	**112**	**56.0**	**200**
>Day 30	**Memory Arm**	Exp 1	40	40.0	6	6.0	54	54.0	100
		Exp 2	39	39.0	8	8..0	53	53.0	100
		**Total**	**79**	**39.5**	**14**	**7.0**	**107**	**53.5**	**200**
	**Exhausted Cl13**	Exp 1	32	31.7	7	6.9	62	61.4	101
		Exp 2	48	48.0	8	8.0	44	44.0	100
		**Total**	**80**	**39.8**	**15**	**7.5**	**106**	**52.7**	**201**

Cd4 CD8$^+$ T cell subset		Monoallelic association SD	Biallelic association SD	No association SD
	**Naïve**	1.4	2.8	1.4
> Day 30	**Memory Arm**	0.7	1.4	0.7
	**Exhausted Cl13**	11.5	0.8	12.3

Statistical analysis (Fisher's 2-tail exact test - combined frequency)	*P*-value	Level	*P*-value	Level	*P*-value	Level
**Naïve vs Memory**	0.837	**ns**	0.840	**ns**	0.688	**ns**
**Naïve vs Exhausted**	0.759	**ns**	0.691	**ns**	0.548	**ns**
**Memory vs Exhausted**	1.000	**ns**	1.000	**ns**	0.920	**ns**

C. Overall ***Cd8*** association to nuclear lamina (number of alleles).

Cd8 CD8$^+$ T cell subset		Associated alleles numbers	%	No association numbers	%	sample size (alleles)	SD
	Exp 1	Exp 1	57	28.5	143	71.5	200

Naïve	Exp 2	Exp 2	61	30.5	139	69.5	200	
	**Total**	**118**	**29.5**	**282**	**70.5**	**400**	**1.4**	
> Day 30 Memory Arm	Exp 1	Exp 1	58	29.0	142	71.0	200	
	Exp 2	Exp 2	54	27.0	146	73.0	200	
	**Total**	**112**	**28.0**	**288**	**72.0**	**400**	**1.4**	
> Day 30 Exhausted Cl13	Exp 1	Exp 1	55	27.2	147	72.8	202	
	Exp 2	Exp 2	61	30.5	139	69.5	200	
	**Total**	**116**	**28.9**	**286**	**71.2**	**402**	**2.3**	

Statistical analysis (Fisher's 2-tail exact test - combined frequency)	*P*-value	Level
**Naïve vs Memory**	0.696	**ns**
**Naïve vs Exhausted**	0.877	**ns**
**Memory vs Exhausted**	0.815	**ns**

D. Monoallelic and biallelic ***Cd8*** association to nuclear lamina (number of cells).

Cd8 CD8^{+} T cell subset		Monoallelic association numbers	%	Biallelic association numbers	%	No association numbers	%	sample size (cells)
	Exp 1	43	43.0	7	7.0	50	50.0	100
	Exp 2	47	47.0	7	7.0	46	46.0	100
	**Total**	**90**	**45.0**	**14**	**7.0**	**96**	**48.0**	**200**
>Day 30 **Memory Arm**	Exp 1	40	40.0	9	9.0	51	51.0	100
	Exp 2	36	36.0	9	9.0	55	55.0	100
	**Total**	**76**	**38.0**	**18**	**9.0**	**106**	**53.0**	**200**
>Day 30 **Exhausted Cl13**	Exp 1	39	38.6	8	7.9	54	53.5	101
	Exp 2	41	41.0	10	10.0	49	49.0	100
	**Total**	**80**	**39.8**	**18**	**9.0**	**103**	**51.2**	**201**

Cd8 CD8^{+} T cell subset	Monoallelic association SD	Biallelic association SD	No association SD
**Naïve**	2.8	0.0	2.8
> Day 30 **Memory Arm**	2.8	0.0	2.8
> Day 30 **Exhausted Cl13**	1.7	1.5	3.2

Statistical analysis (Fisher's 2-tail exact test - combined frequency)	*P*-value	Level	*P*-value	Level	*P*-value	Level
**Naïve vs Memory**	0.187	**ns**	0.581	**ns**	0.368	**ns**
**Naïve vs Exhausted**	0.313	**ns**	0.581	**ns**	0.550	**ns**
**Memory vs Exhausted**	0.759	**ns**	1.000	**ns**	0.765	**ns**

Table S4. Related to Figure 1C. Frequency of ***Pdcd1*** association to nuclear lamina in **Blimp-1 cKO (*Prdm1*$^{-/-}$)** LCMV-specific CD8^{+} T cells during acute infection - DNA-FISH.

A. Overall ***Pdcd1*** association to nuclear lamina (number of alleles).

Pdcd1 CD8^{+} T cell subset	Genotype		Associated alleles numbers	Associated alleles %	No association numbers	No association %	sample size (alleles)	SD
Effector Arm Day 8	***Wild type***	Exp 1	138	69.0	62	31.0	200	
		Exp 2	145	71.1	59	29.9	204	
		Exp 3	-	-	-	-	-	
		**Total**	**304**	**70.1**	**121**	**30.5**	**404**	**1.5**
	***Prdm1*$^{-/-}$**	Exp 1	102	51.0	98	49.0	200	
		Exp 2	102	51.0	98	49.0	200	
		Exp 3	99	49.5	101	50.5	200	
		**Total**	**308**	**54.1**	**262**	**45.9**	**600**	**0.9**

Statistical analysis (Fisher's 2-tail exact test - combined frequency)	*P*-value	Level
**Effector d8 Arm Wt vs *Prdm1*$^{-/-}$**	7.28e-10	***

B. Monoallelic and biallelic ***Pdcd1*** association to nuclear lamina (number of cells).

Pdcd1 CD8^{+} T cell subset	Genotype		Monoallelic association numbers	Monoallelic association %	Biallelic association numbers	Biallelic association %	No association numbers	No association %	sample size (cells)
Effector Arm Day 8	***Wild type***	Exp 1	36	36.0	51	49.0	13	13.0	100
		Exp 2	41	40.2	52	47.1	9	8.8	102
		Exp 3	-	-	-	45.0	-	-	-
		**Total**	**100**	**38.1**	**102**	**51.0**	**29**	**10.9**	**202**
	***Prdm1*$^{-/-}$**	Exp 1	44	44.0	29	29.0	27	17.0	100
		Exp 2	52	52.0	25	25.0	23	23.2	100
		Exp 3	47	47.0	26	26.0	27	27.0	100
		**Total**	**100**	**47.7**	**100**	**26.7**	**100**	**25.7**	**300**

Pdcd1 CD8^{+} T cell subset	Genotype	Monoallelic association SD	Biallelic association SD	No association SD
Effector Arm Day 8	***Wild type***	3.5	0.7	2.8
	***Prdm1*$^{-/-}$**	4.0	2.1	2.3

Statistical analysis (Fisher's 2-tail exact test - combined frequency)	*P*-value	Level	*P*-value	Level	*P*-value	Level
**Effector d8 Arm Wt vs *Prdm1*$^{-/-}$**	0.035445942	*	3.59E-08	***	3.43E-05	***

Table S5. Related to Figure 4. Frequency of ***Pdcd1*** association to nuclear lamina in **Blimp-1 cKO (*Prdm1$^{-/-}$*)** LCMV-specific CD8^{+} T cells during chronic infection - DNA-FISH.

A. Overall ***Pdcd1*** association to nuclear lamina (number of alleles).

Pdcd1 CD8^{+} T cell subset	Genotype		Associated alleles numbers	Associated alleles %	No association numbers	No association %	sample size (alleles)	SD
Effector Cl13 Day 8	*Wild type*	Exp 1	146	70.2	62	29.8	208	
		Exp 2	143	68.7	65	31.2	208	
		Exp 3	135	67.5	65	32.5	200	
		**Total**	**424**	**68.8**	**192**	**31.2**	**616**	**1.4**
	Prdm1$^{-/-}$	Exp 1	120	60.0	80	40.0	200	
		Exp 2	97	49.0	101	51.0	198	
		Exp 3	91	52.9	81	47.1	172	
		**Total**	**308**	**54.1**	**262**	**45.9**	**570**	**5.6**

Statistical analysis (Fisher's 2-tail exact test - combined frequency)	*P*-value	Level
**Effector d8 Cl13 Wt vs *Prdm1$^{-/-}$***	1.877e-7	***

B. Monoallelic and biallelic ***Pdcd1*** association to nuclear lamina (number of cells).

Pdcd1 CD8^{+} T cell subset	Genotype		Monoallelic association numbers	Monoallelic association %	Biallelic association numbers	Biallelic association %	No association numbers	No association %	sample size (cells)
Effector Cl13 Day 8	*Wild type*	Exp 1	44	42.3	51	49.0	9	8.7	104
		Exp 2	45	43.3	49	47.1	10	9.6	104
		Exp 3	45	45.0	45	45.0	10	10.0	100
		**Total**	**134**	**43.5**	**145**	**47.1**	**29**	**9.4**	**308**
	Prdm1$^{-/-}$	Exp 1	63	63.0	37	37.0	17	17.0	100
		Exp 2	55	55.6	21	21.2	23	23.2	99
		Exp 3	57	66.3	17	19.8	12	14.0	86
		**Total**	**175**	**61.6**	**75**	**26.0**	**52**	**18.1**	**285**

Pdcd1 CD8^{+} T cell subset	Genotype	Monoallelic association SD	Biallelic association SD	No association SD
Effector Cl13 Day 8	*Wild type*	1.4	2.0	0.7
	Prdm1$^{-/-}$	5.5	9.6	4.7

Statistical analysis (Fisher's 2-tail exact test - combined frequency)	*P*-value	Level	*P*-value	Level	*P*-value	Level
**Effector d8 Cl13 Wt vs *Prdm1$^{-/-}$***	3.730e-4	***	1.330e-8	***	0.006	**

Table S6. Related to Figure 4. Frequency of ***Ifng*** association to nuclear lamina in **Blimp-1 cKO (*Prdm1$^{-/-}$*)** LCMV-specific CD8^{+} T cells during acute infection - DNA-FISH.

A. Overall ***Ifng*** association to nuclear lamina (number of alleles).

Ifng CD8^{+} T cell subset	Genotype		Associated alleles numbers	%	No association numbers	%	sample size (alleles)	SD
Effector Arm Day 8	***Wild type***	Exp 1	146	73.0	54	27.0	200	
		Exp 2	146	73.0	49	27.0	200	
		**Total**	**292**	**73.0**	**108**	**27.0**	**400**	**0.0**
	Prdm1$^{-/-}$	Exp 1	153	75.7	50	24.3	202	
		Exp 2	148	74.0	61	26.0	200	
		**Total**	**301**	**74.9**	**111**	**25.2**	**402**	**1.2**

Statistical analysis (Fisher's 2-tail exact test - combined frequency)	*P*-value	Level
**Effector d8 Arm Wt vs *Prdm1$^{-/-}$***	5.74e-01	**ns**

B. Monoallelic and biallelic ***Ifng*** association to nuclear lamina (number of cells).

Ifng CD8^{+} T cell subset	Genotype		Monoallelic association numbers	%	Biallelic association numbers	%	No association numbers	%	sample size (cells)
Effector Arm Day 8	***Wild type***	Exp 1	38	38.0	54	54.0	8	8.0	100
		Exp 2	34	34.0	56	56.0	10	10.0	100
		**Total**	**72**	**36.0**	**110**	**55.0**	**18**	**9.0**	**200**
	Prdm1$^{-/-}$	Exp 1	33	32.7	60	59.4	8	7.9	101
		Exp 2	34	34.0	57	57.0	9	9.0	100
		**Total**	**67**	**33.3**	**117**	**58.2**	**17**	**8.5**	**201**

Ifng CD8^{+} T cell subset	Genotype	Monoallelic association SD	Biallelic association SD	No association SD
Effector Arm Day 8	***Wild type***	2.8	1.4	1.4
	Prdm1$^{-/-}$	0.7	1.7	0.8

Statistical analysis (Fisher's 2-tail exact test - combined frequency)	*P*-value	Level	*P*-value	Level	*P*-value	Level
**Effector d8 Arm Wt vs *Prdm1$^{-/-}$***	0.600745959	**ns**	5.46E-01	**ns**	8.62E-01	**ns**

Table S7. Related to Figure 4. Frequency of ***Ifng*** association to nuclear lamina in **Blimp-1 cKO (*Prdm1*$^{-/-}$)** LCMV-specific CD8^{+} T cells during chronic infection - DNA-FISH.

A. Overall ***Ifng*** association to nuclear lamina (number of alleles).

Ifng CD8^{+} T cell subset	Genotype		Associated alleles numbers	Associated alleles %	No association numbers	No association %	sample size (alleles)	SD
Effector Cl13 Day 8	***Wild type***	Exp 1	146	72.3	56	27.7	202	
		Exp 2	139	69.5	61	30.5	200	
		**Total**	**285**	**70.9**	**117**	**29.1**	**402**	**2.0**
	***Prdm1*$^{-/-}$**	Exp 1	150	75.0	50	25.0	200	
		Exp 2	139	69.5	61	30.5	200	
		**Total**	**289**	**72.3**	**111**	**27.8**	**400**	**3.9**

Statistical analysis (Fisher's 2-tail exact test - combined frequency)	*P*-value	Level
**Effector d8 Cl13 Wt vs *Prdm1*$^{-/-}$**	0.696	**ns**

B. Monoallelic and biallelic ***Ifng*** association to nuclear lamina (number of cells).

Ifng CD8^{+} T cell subset	Genotype		Monoallelic association numbers	Monoallelic association %	Biallelic association numbers	Biallelic association %	No association numbers	No association %	sample size (cells)
Effector Cl13 Day 8	***Wild type***	Exp 1	46	45.5	50	49.5	5	5.0	101
		Exp 2	47	47.0	46	46.0	7	7.0	100
		**Total**	**93**	**46.3**	**96**	**47.8**	**12**	**6.0**	**201**
	***Prdm1*$^{-/-}$**	Exp 1	34	34.0	58	58.0	8	8.0	100
		Exp 2	37	37.0	51	51.0	12	12.0	100
		**Total**	**71**	**35.5**	**109**	**54.5**	**20**	**10.0**	**200**

Ifng CD8^{+} T cell subset	Genotype	Monoallelic association SD	Biallelic association SD	No association SD
Effector Cl13 Day 8	***Wild type***	1.0	2.5	1.4
	***Prdm1*$^{-/-}$**	2.1	4.9	2.8

Statistical analysis (Fisher's 2-tail exact test - combined frequency)	*P*-value	Level	*P*-value	Level	*P*-value	Level
**Effector d8 Cl13 Wt vs *Prdm1*$^{-/-}$**	0.033	*	0.195	**ns**	0.145	**ns**

Table S8. Related to Figure S3. Frequency of ***Sell*** association to nuclear lamina in **Blimp-1 cKO (*Prdm1*$^{-/-}$)** LCMV-specific CD8^{+} T cells during acute infection - DNA-FISH.

A. Overall ***Sell*** association to nuclear lamina (number of alleles).

Sell CD8^{+} T cell subset	Genotype		Associated alleles numbers	%	No association numbers	%	sample size (alleles)	SD
Effector Arm Day 8	***Wild type***	Exp 1	159	79.5	41	20.5	200	
		Exp 2	167	81.9	37	18.1	200	
		Exp 3	-	-	-	-	-	
		**Total**	**326**	**80.7**	**78**	**19.3**	**400**	**1.7**
	Prdm1$^{-/-}$	Exp 1	137	63.5	63	36.5	200	
		Exp 2	125	61.6	75	38.4	200	
		Exp 3	107	59.9	93	40.1	200	
		**Total**	**369**	**61.7**	**231**	**38.3**	**600**	**7.5**

Statistical analysis (Fisher's 2-tail exact test - combined frequency)	*P*-value	Level
**Effector d8 Arm Wt vs *Prdm1***$^{-/-}$	6.80e-11	***

B. Monoallelic and biallelic *Sell* association to nuclear lamina (number of cells).

Sell CD8^{+} T cell subset	Genotype		Monoallelic association numbers	%	Biallelic association numbers	%	No association numbers	%	sample size (cells)
Effector Arm Day 8	***Wild type***	Exp 1	27	27.0	66	66.0	7	7.0	100
		Exp 2	37	36.3	65	63.7	0	0.0	102
		Exp 3	-	-	-	-	-	-	-
		**Total**	**64**	**31.6**	**131**	**64.9**	**7**	**3.5**	**202**
	Prdm1$^{-/-}$	Exp 1	35	35.0	51	51.0	14	14.0	100
		Exp 2	47	47.0	39	39.0	14	14.0	100
		Exp 3	61	61.0	23	23.0	16	16.0	100
		**Total**	**143**	**47.7**	**113**	**37.7**	**44**	**14.7**	**300**

Sell CD8^{+} T cell subset	Genotype	Monoallelic association SD	Biallelic association SD	No association SD
Effector Arm Day 8	***Wild type***	7.1	0.7	4.9
	Prdm1$^{-/-}$	13.0	14.0	1.2

Statistical analysis (Fisher's 2-tail exact test - combined frequency)	*P*-value	Level	*P*-value	Level	*P*-value	Level
**Effector d8 Arm Wt vs *Prdm1***$^{-/-}$	0.000431141	***	2.62e-09	***	3.11e-05	***

Table S9. Related to Figure S3. Frequency of ***Sell*** association to nuclear lamina in **Blimp-1 cKO (*Prdm1*$^{-/-}$)** LCMV-specific CD8^{+} T cells during chronic infection - DNA-FISH.

A. Overall ***Sell*** association to nuclear lamina (number of alleles).

Sell CD8^{+} T cell subset	Genotype		Associated alleles numbers	Associated alleles %	No association numbers	No association %	sample size (alleles)	SD
Effector Cl13 Day 8	***Wild type***	Exp 1	171	82.2	37	17.8	208	
		Exp 2	189	86.7	29	13.3	218	
		Exp 3	170	85.0	30	15.0	200	
		**Total**	**530**	**84.6**	**96**	**15.4**	**626**	**2.3**
	***Prdm1*$^{-/-}$**	Exp 1	127	63.5	73	36.5	200	
		Exp 2	122	61.6	76	38.4	198	
		Exp 3	103	59.9	69	40.1	172	
		**Total**	**352**	**61.7**	**218**	**38.3**	**570**	**1.8**

Statistical analysis (Fisher's 2-tail exact test - combined frequency)	*P*-value	Level
**Effector d8 Cl13 Wt vs *Prdm1*$^{-/-}$**	1.492e-19	***

B. Monoallelic and biallelic *Sell* association to nuclear lamina (number of cells).

Sell CD8^{+} T cell subset	Genotype		Monoallelic association numbers	Monoallelic association %	Biallelic association numbers	Biallelic association %	No association numbers	No association %	sample size (cells)
Effector Cl13 Day 8	***Wild type***	Exp 1	33	31.7	69	66.3	2	1.9	104
		Exp 2	25	22.9	82	75.2	2	1.8	104
		Exp 3	28	28.0	71	71.0	1	1.0	100
		**Total**	**86**	**27.6**	**222**	**70.9**	**5**	**1.6**	**313**
	***Prdm1*$^{-/-}$**	Exp 1	49	49.0	39	39.0	12	12.0	100
		Exp 2	52	52.5	35	35.4	12	12.1	99
		Exp 3	39	45.3	32	37.2	15	17.4	86
		**Total**	**140**	**49.0**	**106**	**37.2**	**39**	**13.9**	**285**

Sell CD8^{+} T cell subset	Genotype	Monoallelic association SD	Biallelic association SD	No association SD
Effector Cl13 Day 8	***Wild type***	4.4	4.4	0.5
	***Prdm1*$^{-/-}$**	3.6	1.8	3.1

Statistical analysis (Fisher's 2-tail exact test - combined frequency)	Monoallelic *P*-value	Level	Biallelic *P*-value	Level	No association *P*-value	Level
**Effector d8 Cl13 Wt vs *Prdm1*$^{-/-}$**	5.772e-8	***	3.137e-16	***	5.727e-9	***

Table S10. Related to Figure S4. Frequency of ***Pdcd1*** association to nuclear lamina in Dnmt3a cKO (*Dnmt3a*$^{-/-}$) LCMV-specific CD8^{+} T cells during acute infection - DNA-FISH.

A. Overall ***Pdcd1*** association to nuclear lamina (number of alleles).

Pdcd1 CD8^{+} T cell subset	Genotype		Associated alleles numbers	Associated alleles %	No association numbers	No association %	sample size (alleles)	SD
Effector Arm Day 8	***Wild type***	Exp 1	134	67.0	66	33.0	200	
		Exp 2	142	71.0	58	29.0	200	
		Exp 3	141	70.5	59	29.5	200	
		**Total**	**417**	**69.5**	**183**	**30.5**	**600**	**2.2**
	Dnmt3a$^{-/-}$	Exp 1	104	52.0	96	48.0	200	
		Exp 2	118	59.0	82	41.0	200	
		Exp 3	95	47.5	105	52.5	200	
		**Total**	**317**	**52.8**	**283**	**47.2**	**600**	**5.8**

Statistical analysis (Fisher's 2-tail exact test - combined frequency)	*P*-value	Level
**Effector d8 Cl13 Wt vs *Dnmt3a*$^{-/-}$**	4.089e-9	***

B. Monoallelic and biallelic ***Pdcd1*** association to nuclear lamina (number of cells).

Pdcd1 CD8^{+} T cell subset	Genotype		Monoallelic association numbers	Monoallelic association %	Biallelic association numbers	Biallelic association %	No association numbers	No association %	sample size (cells)
Effector Arm Day 8	***Wild type***	Exp 1	42	42.0	46	46.0	12	12.0	100
		Exp 2	38	38.0	52	52.0	10	10.0	100
		Exp 3	43	43.0	49	49.0	8	8.0	100
		**Total**	**123**	**41.0**	**147**	**49.0**	**30**	**10.0**	**300**
	Dnmt3a$^{-/-}$	Exp 1	50	50.0	27	46.0	23	12.0	100
		Exp 2	52	52.0	33	33.0	15	15.0	100
		Exp 3	49	49.0	23	23.0	28	28.0	100
		**Total**	**151**	**50.3**	**83**	**27.7**	**66**	**22.0**	**300**

Pdcd1 CD8^{+} T cell subset	Genotype	Monoallelic association SD	Biallelic association SD	No association SD
Effector Arm Day 8	***Wild type***	2.6	3.0	2.0
	Dnmt3a$^{-/-}$	1.5	5.0	6.6

Statistical analysis (Fisher's 2-tail exact test - combined frequency)	*P*-value	Level	*P*-value	Level	*P*-value	Level
**Effector d8 Cl13 Wt vs *Dnmt3a*$^{-/-}$**	0.027	*	1.067e-7	***	8.375e-5	**

Table S11. Related to Figure S4. Frequency of ***Sell*** association to nuclear lamina in Dnmt3a cKO (*Dnmt3a*$^{-/-}$) LCMV-specific CD8^{+} T cells during acute infection - DNA-FISH.

A. Overall *sell* association to nuclear lamina (number of alleles).

Sell CD8^{+} T cell subset	Genotype		Associated alleles numbers	%	No association numbers	%	sample size (alleles)	SD
Effector Arm Day 8	***Wild type***	Exp 1	165	82.5	35	17.5	200	
		Exp 2	167	83.5	33	16.5	200	
		Exp 3	175	87.5	25	12.5	200	
		**Total**	**507**	**84.5**	**93**	**15.5**	**600**	**2.6**
	Dnmt3a$^{-/-}$	Exp 1	113	56.5	87	43.5	200	
		Exp 2	139	68.8	63	31.2	202	
		Exp 3	116	58.0	84	42.0	200	
		**Total**	**368**	**61.1**	**234**	**38.9**	**602**	**6.7**

Statistical analysis (Fisher's 2-tail exact test - combined frequency)	*P*-value	Level
**Effector d8 Cl13 Wt vs *Dnmt3a*$^{-/-}$**	4.277e-20	***

B. Monoallelic and biallelic *Sell* association to nuclear lamina (number of cells).

Sell CD8^{+} T cell subset	Genotype		Monoallelic association numbers	%	Biallelic association numbers	%	No association numbers	%	sample size (cells)
Effector Arm Day 8	***Wild type***	Exp 1	29	29.0	68	68.0	3	3.0	100
		Exp 2	21	21.0	73	73.0	6	6.0	100
		Exp 3	23	23.0	76	76.0	1	1.0	
		**Total**	**73**	**24.3**	**217**	**72.3**	**10**	**3.3**	**300**
	Dnmt3a$^{-/-}$	Exp 1	41	41.0	36	36.0	23	23.0	100
		Exp 2	41	40.6	49	48.5	11	10.9	101
		Exp 3	46	46.0	35	35.0	19	19.0	100
		**Total**	**128**	**42.5**	**120**	**39.8**	**53**	**17.6**	**301**

Sell CD8^{+} T cell subset	Genotype	Monoallelic association SD	Biallelic association SD	No association SD
Effector Arm Day 8	***Wild type***	4.2	4.0	2.5
	Dnmt3a$^{-/-}$	3.0	7.5	6.2

Statistical analysis (Fisher's 2-tail exact test - combined frequency)	*P*-value	Level	*P*-value	Level	*P*-value	Level
**Effector d8 Cl13 Wt vs *Dnmt3a*$^{-/-}$**	2.767e-6	***	8.714e-16	***	6.236e-9	***

References

1. Ghoneim, H.E., Fan, Y., Moustaki, A., Abdelsamed, H.A., Dash, P., Dogra, P., Carter, R., Awad, W., Neale, G., Thomas, P.G., and Youngblood, B. (2017). De Novo Epigenetic Programs Inhibit PD-1 Blockade-Mediated T Cell Rejuvenation. Cell *170*, 142-157 e119. 10.1016/j.cell.2017.06.007.
